# Influences of Basis Set and Electronic Exchange-Correlation on Low-Frequency Vibrations and Stability of Paracetamol Polymorphs

**DOI:** 10.1021/acs.jctc.5c00599

**Published:** 2025-10-01

**Authors:** Huanyu Zhou, Giuseppe Mallia, Nicholas M. Harrison

**Affiliations:** Department of Chemistry, 4615 Imperial College London, White City Campus, 80 Wood Lane, London W12 0BZ, United Kingdom

## Abstract

In molecular crystals, many solid state phase transitions can be attributed to the entropy of intermolecular vibrations at low frequencies, where the weak noncovalent interactions dominate, and simulations based on density functional theory (DFT) suffer from relatively large errors. In this work, the precision of two critical computational parameters are evaluated, namely the choice of local basis set and (DFT-based) Hamiltonian. Each is documented in detail for the vibrational properties and thermodynamic stability of two polymorphs of paracetamol, which are chosen due to the representative chemical interactions and the good availability of high-quality reference data. Comparisons are made with experimentally measured low-temperature geometries, Raman spectra, and temperature–pressure phase diagrams. These results highlight the substantial influences of basis set, revealing the importance of both the cardinality (i.e., ζ) and the diffuseness; the failure to account for either factor might lead to unexpected results. The features of low-frequency vibrations that most significantly affect the relative stability of molecular crystals are identified and shown to be captured when the triple-ζ def2-TZVP basis set is adopted; on the contrary, widely used double-ζ basis sets are shown to be inadequate. The improvement attributed to the inclusion of Fock exchange is revealed to be marginal, especially with triple-ζ basis sets. By systematically improving levels of precision, the current work disentangles competing sources of errors impeding accurate crystal structure predictions and polymorph energy rankings. This facilitates the predictability and reliability of DFT in thermodynamic modeling of molecular crystals.

## Graphical abstract

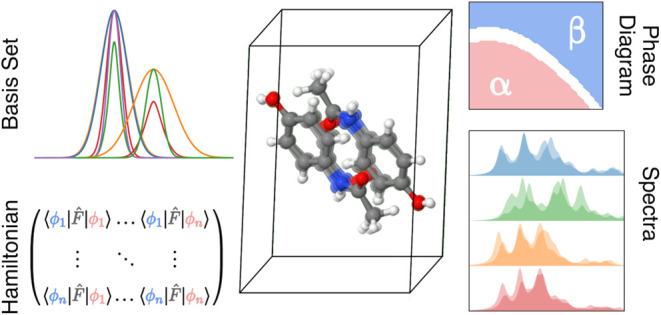

## Introduction

Molecular crystals are the common crystalline form of organic compounds, where the constituent molecules are packed into ordered motifs within a periodic lattice. The physicochemical properties of the constituent molecules are perturbed by weak intermolecular interactions such as London dispersion and hydrogen bonding. A large percentage of compounds of biological and pharmaceutical importance form molecular crystals.^1^ As a common feature, it is estimated that at least 50% of organic molecular crystals exhibit polymorphism,^2,3^ i.e., the existence of multiple crystalline phases of the same compound. This phenomenon can be attributed to the weak noncovalent interactions between molecules and the consequent subtle energy differences between the different packing motifs, making their relative stability highly sensitive to various environmental factors including humidity, temperature and pressure. As a consequence, solid state phase transitions can easily be triggered, leading to unexpected variations in physicochemical properties and potential influences on pharmaceutical manufacturability, reactivity, and bioavailability.^1,4^ A thorough understanding of the interactions present in chemically inhomogeneous molecular crystals is necessary to be able to design, synthesize and chemically process polymorphs with desired properties. However, as suggested by the latest crystal structure prediction (CSP) blind test,^5^ this remains challenging for state-of-the-art atomic-scale simulations, due to the peculiar nature of molecular crystals and the difficulties involved in controlling the delicate interplay of multiple sources of error arising from each adopted level of theory.

The role of lattice vibrations is non-negligible when ranking the polymorph energies of molecular crystals, as previously suggested in both theoretical and experimental studies.^6–8^ Low-frequency vibrations usually involve collective molecular motions, and so their frequencies and associated eigenvectors provide detailed information about the arrangement pattern of molecules within the lattice. As a consequence, low-frequency spectroscopy, such as Raman and terahertz time-domain spectra, has become important techniques used to probe and discriminate polymorphs of molecular crystals.^7,9,10^ Low-frequency lattice vibrations can be thermally activated under ambient conditions, leading to a wide range of phenomena, including lattice expansion, phase transition, and charge and heat transport.^11–14^

From a theoretical perspective, multiple challenges are encountered when modeling intermolecular vibrations at low frequencies with density functional theory (DFT), regardless of its widespread successful applications in treating metallic, ionic and covalent-bonded systems. As an inherent drawback of DFT, long-range London dispersion interactions are neglected, weakening the computed bindings between the constituent molecules.^15,16^ Nevertheless, probably due to error cancellations, limited influences of neglected London dispersions on vibrational frequencies have been reported in several previous studies.^11,13,17^ Basis set incompleteness errors may also substantially influence the results obtained, especially for atomic orbital basis sets that are commonly adopted in quantum chemistry, where the finite Gaussian-type orbitals give rise to the well-documented basis set superposition error (BSSE), leading to the overbinding between molecules and the overestimation of intermolecular vibrational frequencies.^15,18–20^ Several studies have also stressed the importance of the Hamiltonian adopted, in which including nonlocal Fock exchange or the second-order Møller–Plesset perturbation can improve descriptions of electronic correlation and London dispersions, and therefore the prediction of both geometry and lattice vibrations^16,20–22^ Though, ideally, errors induced by basis set and Hamiltonian can be reduced monotonically as the level of theory improves, this inevitably increases the computational load. Compared with many other materials, molecular crystals are generally characterized by larger unit cells and reduced symmetry. This necessitates an efficient approach that balances the cost and precision of simulations and is particularly important for computationally demanding studies on vibrational and thermodynamic properties.^14,23,24^

As a widely used pharmaceutical, paracetamol is a prototype of both scientific and commercial importance. Therefore, a broad range of experimental studies have been performed to resolve its crystal structures,^25–27^ Raman spectra,^9,10^ and phase stability,^28^ providing high-quality reference data that are critical for evaluating the accuracy of theoretical methods. Various noncovalent interactions of distinct properties are known to exist within the lattice, including London dispersion, π–π stackings, and hydrogen bonds, and these are representative of those commonly present in molecular crystals. Such nonuniformity gives rise to peculiar phenomena, such as the rapid growth of specific facets^29,30^ or the emergence of metastable phases,^31^ where very limited knowledge of the underlying thermodynamic and kinetic mechanisms is available. As previously introduced, these interactions are also challenging for CSP and predictions of vibrational properties. Subtle lattice energy differences as small as 0.9 kJ/mol between the observed and the hypothetical phases are reported.^32^ Strong anharmonicity is identified in both the low-frequency^6,11,33^ and high-frequency phonon modes.^12^ The sublimation enthalpies obtained with different numerical models also vary by 23.1 kJ/mol.^21^

In the current work, the geometry, intermolecular vibrations, and relative stability of paracetamol are benchmarked in detail to evaluate the influences of basis set and Hamiltonian, which are compared to the published experimental measurements. Given the wide range of basis sets and exchange-correlation functionals available, an exhaustive assessment might be infeasible. Therefore, the Karlsruhe “def2” basis sets and their variants, namely def2-SVP, def2-TZVP and pob-TZVP,^34–36^ are adopted in an attempt to systematically document the impact of cardinality (i.e., ζ) and diffuseness of basis sets. The smaller and frequently adopted Pople 6–31G** basis set^37^ is also included for comparison. The plane-wave basis set offers a potentially complete reference. However, it might be restrictive for the inhibitive cost in Fock exchange calculations, necessitating the use of atomic basis sets that obtain comparable results and are affordable for Fock exchange. To compare the predictions of DFT and Hartree–Fock (HF) Hamiltonians, results based on the semilocal, generalized gradient approximated Perdew–Burke–Ernzerhof functional (GGA-PBE)^38^ are compared with those obtained with its global hybrid variant with 25% Fock exchange, PBE0.^39^ All of the benchmark parameters identified here are widely adopted in practical CSP research, while representative intermolecular interactions, such as London dispersions, π–π stackings and hydrogen bonds, are present in the targeted structures with moderate complexity, which ensures that the findings of this study can directly facilitate the parameter choices when ranking the free energies of unknown polymorphs.

## Methodology

DFT simulations based on the linear combination of atomic orbitals (LCAO) method are performed with CRYSTAL23,^40,41^ while those based on the plane-wave projected augmented wave (PAW) method are performed with Quantum Espresso 7.3.1.^42,43^ For LCAO, the GGA-PBE functional^38^ and the hybrid PBE0 functional with 25% Fock exchange^39^ are used for electron exchange and correlation; while for PAW, only GGA-PBE is used due to the prohibitive computational resources required for Fock exchange in plane-wave implementations. In contrast to all-electron atomic basis sets, effective core pseudopotentials based on PAW and GGA-PBE are used with plane-wave basis sets.^44^ Their influences are negligible in the presented results, probably due to the dominant noncovalent London dispersion within the lattice, which is mainly attributed to valence electrons. To account for London dispersions, the *a posteriori* D3 correction of Grimme et al.^45^ is used with the Becke–Johnson damping (D3-BJ)^46^ and three-body terms, which yields a good agreement with both experimental and theoretical references, according to several benchmarking studies on organic molecular crystals.^13,20,47^ D3-BJ is available for both PBE0 and PBE, facilitating the comparison between the DFT and the hybrid DFT-HF Hamiltonians. The pure HF Hamiltonian is omitted since D3-BJ is not available. Double-ζ basis sets (6–31G**^37^ and def2-SVP^34,35^ ) and triple-ζ basis sets (pob-TZVP^36^ and def2-TZVP^34,35^ ) are benchmarked. These basis sets are commonly adopted in studying molecular crystals,^15,20,48,49^ making them good candidates for the benchmarking study aimed for polymorph energy rankings. Other general purpose atomic basis sets were tested at the preparation stage of this study, and a detailed discussion is available in Section S2.1. The geometrical counterpoise (gCP) method^49,50^ is applied to correct BSSE of atomic basis sets. For PAW, the cutoff energies of the electronic wave function and density are set to 100 and 400 Ry, respectively, ensuring the convergence of at least 3.3 × 10^–6^ Ry per atom. In all simulations, the 4 × 4 × 2 Monkhorst–Park grid is adopted to sample the Brillouin zone of both form I and form II crystals. The total energy per cell during the self-consistency step is converged to 10^–9^ Hartree. Geometries are fully optimized to high numerical precision in order to eliminate imaginary modes during phonon calculations with energy gradients smaller than 1 × 10^–4^ Hartree/Bohr. More details are available in the Supporting Information.

Phonon frequencies are obtained with the finite displacement method as implemented in CRYSTAL23 and phonopy.^51,52^ In both cases, the space group symmetry is fully exploited to minimize the number of displacements. The “2-step” central difference formalism is used to compute the Hessian matrix at Γ, which is sufficient for Raman spectra.^11,13^ The dielectric constants, Born effective charge, and Raman intensities based on LCAO are computed with the coupled-perturbed Hartree–Fock/Kohn–Sham (CPHF/KS) method as implemented in CRYSTAL23. For PAW, the dielectric constants and Born effective charge are computed with the density functional perturbation theory (DFPT) as implemented in Quantum Espresso 7.3.1, with the squared residual of the linear response potential within 10^–15^ Ry^2^. Raman intensities are computed with phonopy-spectroscopy^53^ with the far-from-resonance approximation. For comparison with experimental Raman spectra, a factor to take into account the angular frequency ω_0_ of the incoming laser and the temperature *T* is introduced,^54^ which, for a Raman-active mode *m*, gives

1 $$I_{m}=\frac{{\left(\omega_{0}-\omega_{m}\right)}^{4}}{c^{4}}\frac{\hbar }{\omega_{m}}\left(\frac{1}{\exp\left(\frac{\hbar \omega_{m}}{k_{B}T}\right)-1}+1\right)I_{m}^{\prime }$$

where *c* is the speed of light in vacuum and *I*_*m*_^′^ ∝ (**α**/∂ ***e***_***m***_)^2^, which is the Raman intensity obtained from the polarizability tensor **α** and the mode eigenvector ***e*_*m*_**. In this work, the unpolarized polycrystalline spectra of form I and II paracetamol crystals are reported with ω_0_ = 3540.70 THz (i.e., λ = 532 nm) and *T* = 300 K.^10^ All Raman peaks are uniformly broadened with Lorentzian function and full width at half-maximum (FWHM) of 10 cm^–1^ to mimic the finite experimental resolution.

The equivalent phonon modes obtained by LCAO and PAW are identified by the dot product of their eigenvectors ***e***_LCAO_· ***e***_PAW/PBE_, where, under idealized conditions, the dot product between the equivalent normal modes is equal to one, and those between the nonequivalent modes are zero. In practice, the sum of dot products between all eigenvectors is maximized as a linear-sum-assignment problem with CRYSTALpytools^55,56^

2 $$\max\sum_{i}^{3N-3}\sum_{j}^{3N-3}X_{\mathrm{ij}}e_{\mathrm{PAW}/\mathrm{PBE}}\cdot e_{\mathrm{LCAO}}$$

where *N* is the number of atoms, 3*N* – 3 is the number of nontranslational modes and ***X***_***ij***_ is the boolean matrix whose element *x*_*ij*_ = 1 when the *j*th mode of LCAO is assigned to the *i*th mode of PAW. To reduce the dimensionality of the problem, eq 2 is applied to modes with the same symmetry.

The Gibbs free energy *F* is computed with CRYSTALpytools, which can be expressed as the sum of the DFT-based electronic energy *E*_DFT_ and the vibrational contributions

3 $$F=E_{\mathrm{DFT}}+\sum_{i,q}\frac{1}{2}\hbar \omega_{i,q}+\sum_{i,q}k_{B}T\ln\left[1-\exp\left(-\frac{\hbar \omega_{i,q}}{k_{B}T}\right)\right]+\mathrm{pV}$$

*k*_B_ is the Boltzmann constant. *T* and *p* are the temperature and pressure, respectively. ω is the angular vibrational frequency. The summation is taken over the vibrational mode *i* and the point ***q*** in reciprocal space. In this work, phonon dispersions on a 4 × 4 × 2 Γ-centered Monkhorst–Park grid are obtained on by interpolating the Hessian matrix with phonopy. The direct space approach for phonon dispersions requires a supercell, which is computationally unfeasible.

## Results and Discussion

In this work, two common crystalline phases of paracetamol are considered, namely the monoclinic form I (FI) and the orthorhombic form II (FII), for benchmarking tests (Table S2). FI is the thermodynamically most stable phase under ambient conditions and the most common form observed. It is characterized by corrugated hydrogen bond networks of NH···OH and OH···O=C stacked along the [010] direction (i.e., *b* of Figure 1a), which increase friction of the interlayer slip, causing difficulties in downstream processing, such as tableting.^4^ In the metastable FII phase, nearly planar hydrogen bond networks are stacked with shorter interlayer distances (∼3.57 Å, compared to ∼4.48 Å of FI) along the [010] direction (i.e., *b* of Figure 1b). Previous theoretical studies have revealed the subtle energy difference of 0.29–8.2 kJ/mol (per mole of paracetamol molecules, C_8_H_9_NO_2_) between FI and FII under ambient conditions, and lattice vibrations play a critical role.^12,32^ These factors stress the peculiar nature of molecular crystal polymorphism, which is extremely sensitive to the structural and chemical properties of constituting molecules, as well as environmental conditions. Therefore, it is necessary to thoroughly document the effect of the main computational approximations (the Hamiltonian and the basis set) on the computed thermodynamic properties.

**Figure 1:**
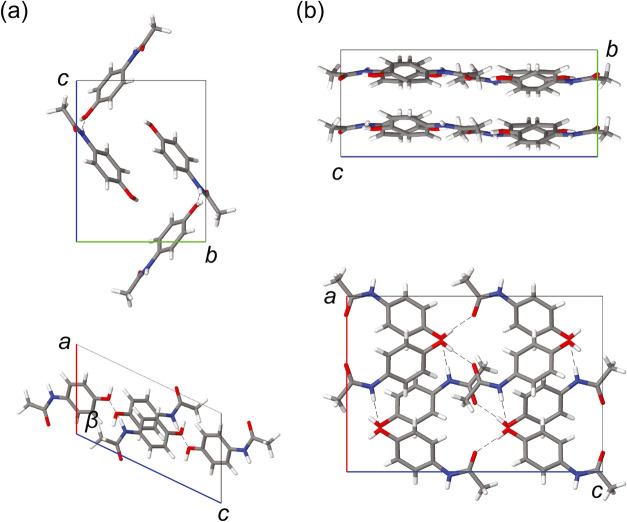
Unit cells of (a) form I and (b) form II paracetamol crystals. White, gray, blue and red colors respectively denote hydrogen, carbon, nitrogen and oxygen atoms. Hydrogen bonds are denoted by the black dashed lines.

### Optimized Geometries

As a critical prerequisite, geometry significantly influences vibrational spectra and thermodynamic functions. The deviation of the predicted geometry from that observed is important for evaluating the potential energy surface described by Hamiltonian and basis set. In Table 1, optimized geometries of FI and FII are compared with those deduced from low-temperature diffraction measurements at 20 K.^25,26^ It should be noted that, in all cases, optimization is performed on the athermal energy surface neglecting contributions of lattice vibrations requiring phonon calculations at each optimization step, which is beyond the capacity of modern high-performance computers for these systems. Studies on small molecular crystals such as CO_2_ phase I and ice phase Ih found moderate volume expansions of no more than 3.3% at 0 K by including vibrational contributions and this expansion may be expected to decrease with the mass of molecules.^21,57^ Therefore, considering the mass of paracetamol, the influence of vibration on geometry is likely to be negligible.

**Table 1:** Optimized Geometries of Forms I and II Paracetamol Crystals

polymorph	Hamiltonian	basis set	volume (Å^3^)^a^	RMSD (Å)
form I	PBE0	6–31G**	782.76 (5.61)	0.16
		def2-SVP	793.89 (7.11)	0.18
		pob-TZVP	735.73 (−0.73)	0.32
		def2-TZVP	752.74 (1.56)	0.06
	PBE	6–31G**	788.68 (6.41)	0.22
		def2-SVP	817.53 (10.31)	0.28
		pob-TZVP	738.81 (−0.32)	0.36
		def2-TZVP	747.16 (0.81)	0.16
		PAW	751.50 (1.40)	0.14
form II	PBE0	6–31G**	1522.59 (5.65)	0.15
		def2-SVP	1551.47 (7.66)	0.18
		pob-TZVP	1453.96 (0.89)	0.13
		def2-TZVP	1458.32 (1.19)	0.07
	PBE	6–31G**	1540.59 (6.90)	0.19
		def2-SVP	1603.18 (11.25)	0.25
		pob-TZVP	1465.10 (1.66)	0.15
		def2-TZVP	1455.89 (1.03)	0.11
		PAW	1466.95 (1.79)	0.11

a The percentage error with respect to experimental data (detailed in Table S2) is reported in parentheses.

Compared to the experimental data (*V*_exp_), predicted volumes (*V*_DFT_) are generally overestimated, except *V*_DFT_ of FI by pob-TZVP, which is underestimated by less than 1%. All *V*_DFT_ computed using double-ζ basis sets deviate from *V*_exp_ by over 5%, and the smaller 6–31G** outperforms the larger def2-SVP. This probably originates from the more diffusive 2p orbitals of the hydrogen bond acceptor (O) in 6–31G**, which better describes the increased electron densities at long-range, i.e., within the NH···OH and OH···O=C hydrogen bonds. A better agreement with *V*_exp_ is observed as the cardinality of basis set increases. The deviation of *V*_DFT_ is reduced to approximately 1.5% with triple-ζ basis sets, which also produces results close to those obtained by the nearly complete and BSSE-free plane-wave PAW method. Though pob-TZVP leads to a *V*_DFT_ that is closer to that observed than the larger def2-TZVP, the optimized lattice parameters (Table S3) reveal a distinctly opposite trend, suggesting that the nominal better agreement for *V*_DFT_ originates from a fortuitous and anisotropic cancellation of errors.

The percentage errors of the lattice parameters in Table S3 show a clear anisotropy. For both FI and FII, the largest deviation is observed for *b*. The lattice vector *b* is the stacking direction of the hydrogen bond networks (Figure 1), where the weaker dispersion interactions dominate. As an inherited drawback of DFT, descriptions of long-range dispersion interactions largely depend on the *a posteriori* method adopted, which has been thoroughly discussed elsewhere^13,15^ and a detailed examination of this is beyond the scope of the current work. Nevertheless, this illustrates how the packing motifs within the lattice alter the dominant interactions among molecules, which consequently influence the outcomes, highlighting the importance of correctly reproducing the internal atomic coordinates. To compare the similarity of the packing patterns between the predicted and experimental structures, a distance-based algorithm^58^ is adopted, and the root-mean-square-deviation (RMSD) are summarized in Table 1. Following a similar trend as aforementioned, among all atomic basis sets, the def2-TZVP basis set achieves the best agreement in packing similarity with low-temperature measurements, as characterized by the lowest RMSD, while the def2-SVP basis set has the highest RMSD. In particular, the RMSD of def2-TZVP/PBE is comparable to that of PAW/PBE, indicating the well-converged performance of the medium-sized triple-ζ basis set for FI and FII.

Though the plane-wave basis set is orthogonal and BSSE-free, its cost becomes prohibitive when Fock exchange is computed, e.g., for hybrid functionals. The self-interaction error of nonlocalized density functionals might become significant when describing many-body interactions within molecular clusters and crystals, as revealed in previous benchmarking studies.^2,59,60^ However, results of this study suggest limited improvements in geometry prediction when the hybrid functional PBE0 is used with def2-TZVP. Considering the well-converged performances from def2-TZVP to plane-wave, negligible differences might be expected between the nearly complete PAW/PBE and PAW/PBE0 methods. In comparison, observable improvements are reported in the geometries predicted by the smaller double-ζ basis sets, where errors in *V*_DFT_ are reduced by over 1% and RMSDs are reduced by 0.04–0.1 Å. These improvements, however, fail to counterbalance the errors of poorly converged basis sets, leading to worse geometries than those predicted with larger basis sets and the less expensive PBE functional.

From the above, we can conclude that the largest LCAO basis set benchmarked, def2-TZVP, provides the nearly complete basis set convergence for the current purpose, emphasizing the importance of the cardinality of the basis set adopted in these systems. Comparisons between basis sets with the same cardinality highlight the diffuseness of valence orbitals, where the more diffusive valence orbitals probably offer better descriptions of noncovalent interactions. Furthermore, including Fock exchange has very limited improvements that become negligible as cardinality and diffuseness of the basis set increase.

### Low-Frequency Raman Spectra and Lattice Vibrations

The low-frequency lattice vibrations of molecular crystals are dominated by weak intermolecular interactions, such as dispersion and hydrogen bonding, which are sensitive to the packing motifs of molecules in the lattice. It has been demonstrated in the experimental studies of Roy et al.^9^ and Nanubolu and Burley,^10^ that the low-frequency region contains key Raman spectral features that distinguish FI and FII, while the peak positions at higher frequencies (dominated by intramolecular interactions) are almost identical. To assess the performance of basis set and Hamiltonian in describing the collective motions of molecules in the lattice, the simulated Raman spectra of FI and FII ranging from 0 to 250 cm^–1^ are compared with the published experimental data^10^ in Figure 2. Although it has been widely reported that spectra based on experimental lattice usually agree better with the reference measured at finite temperatures than the relaxed, probably due to thermal expansions,^11,13,61^ such data might not be available for practical CSP workflows. Therefore, here all spectra are computed with the optimized geometries reported in the previous section.

**Figure 2:**
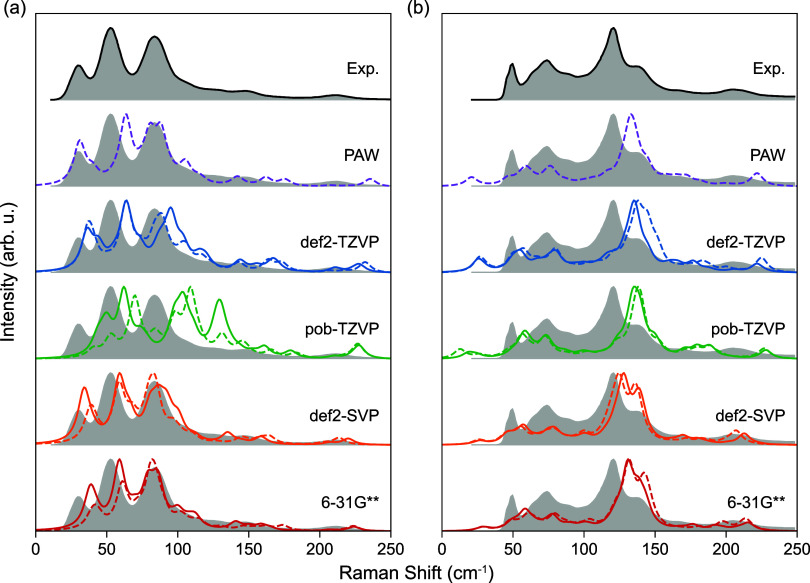
Experimental and calculated low-frequency Raman spectra of (a) form I and (b) form II paracetamol crystals between 0 and 250 cm^–1^. Spectra in solid lines are obtained by PBE0, while those in dashed lines are obtained by PBE. Intensities of are normalized to the highest peak of each plot. For comparison, the experimental spectra are replicated as gray shaded areas. Experimental spectra adapted with permission from ref 10. Copyright 2012 American Chemical Society.

For both FI and FII, the spectra predicted by def2-TZVP agree well with PAW, again suggesting that the local basis set is near convergence. The prominent blue-shifts of Raman peaks probably originate from the neglected thermal expansion under experimental temperature (300 K), where the reduced intermolecular interaction softens phonon vibrations. Inconsistencies are observed for the spectra obtained with the pob-TZVP basis set, where peak positions of FI are significantly shifted by ∼50 cm^–1^, as a result of the poor geometry prediction, while predictions of FII are comparable to those of def2-TZVP. Such inconsistencies originate from the only partially corrected BSSE, which becomes increasingly significant with FI, probably due to π–π stacking along the lattice vector *b* (Figure S3). This finding indicates that, for molecular crystals with lower symmetry and density, the transferability of solid-state basis sets adapted from their molecular counterparts might be questionable. London dispersions that dominate within the lattice probably require more diffusive valence and polarization orbitals. Despite poor geometry predictions, Raman spectra from double-ζ basis sets exhibit notable agreement with the experiment. This phenomenon is likely attributable to the delicate error cancellation, where the overestimated volumes soften the phonon and red-shift the spectra. Considering the ambiguous and inhomogeneous interactions within molecular crystals, relying on the counterbalance between geometry and thermal effects might not be practical for a predictive CSP workflow.

Compared to the significant errors introduced by incomplete basis sets, the inclusion of Fock exchange only gives rise to marginal variations, as indicated in Figure 2. For FI, the largest discrepancy lies in the intensity of the molecule rotation mode around 83 cm^–1^, while for FII, PBE0 red-shifts the shoulder peak associated with the rotating methyl group around 136 cm^–1^. For both modes, increased discrepancies between their numerical potential energy surface and the harmonically approximated one are observed (Figure S1), indicating that Fock exchange could more significantly influence modes with stronger anharmonicity.

Though comparisons between theoretical and experimental Raman spectra provide reliable and effective benchmarks of vibrational properties, the assignment of experimental Raman peaks could be ambiguous, since a Raman peak might originate from multiple overlapped Raman-active modes.^13^ In addition, Raman-inactive modes are absent from such comparisons. For a complete benchmark, as proposed in eq 2, all nontranslational phonon modes from LCAO are unambiguously sorted with reference to PAW/PBE results, since the latter achieves the best agreement with experimental data. The distribution of the dot products, ***e***_PAW/PBE_· ***e***_LCAO_, is illustrated in Figure 3a for the intermolecular modes (0 to 250 cm^–1^) of FI. The frequencies obtained by LCAO and PAW/PBE are compared in Figure 3b,c. Similarly, data of FII is visualized in Figure 4.

**Figure 3:**
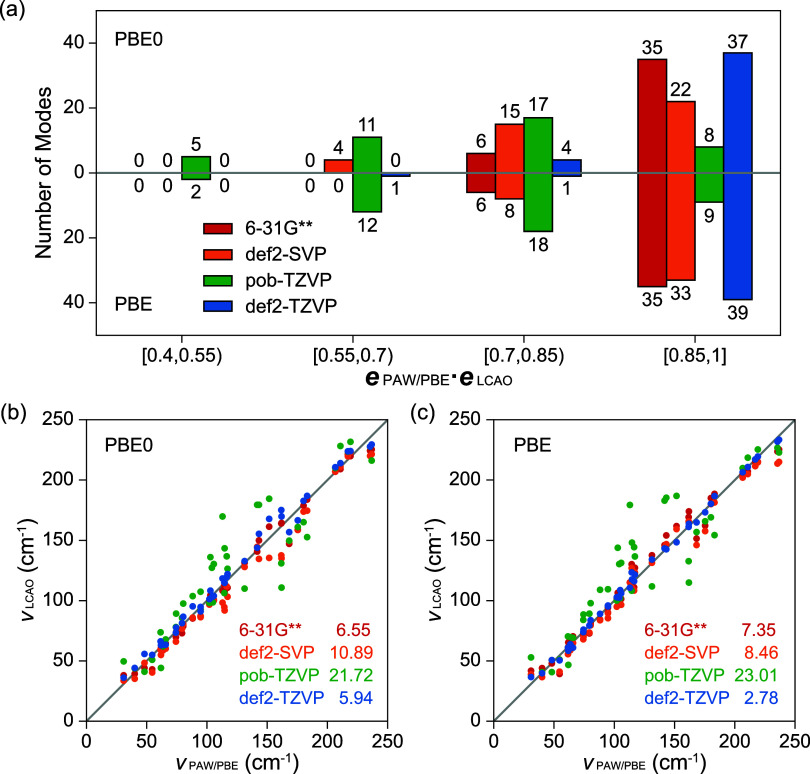
Similarities of intermolecular vibrational modes (0–250 cm^–1^) of form I paracetamol. (a) Distributions of dot products between mode eigenvectors obtained by PAW/PBE (***e***_PAW/PBE_) and those by LCAO/PBE­(PBE0) (***e***_LCAO_). (b, c) Phonon frequencies calculated by LCAO/PBE­(PBE0) (ν_LCAO_) as functions of phonon frequencies by PAW/PBE (ν_PAW/PBE_). Root-mean-square-deviations are annotated with legends.

**Figure 4:**
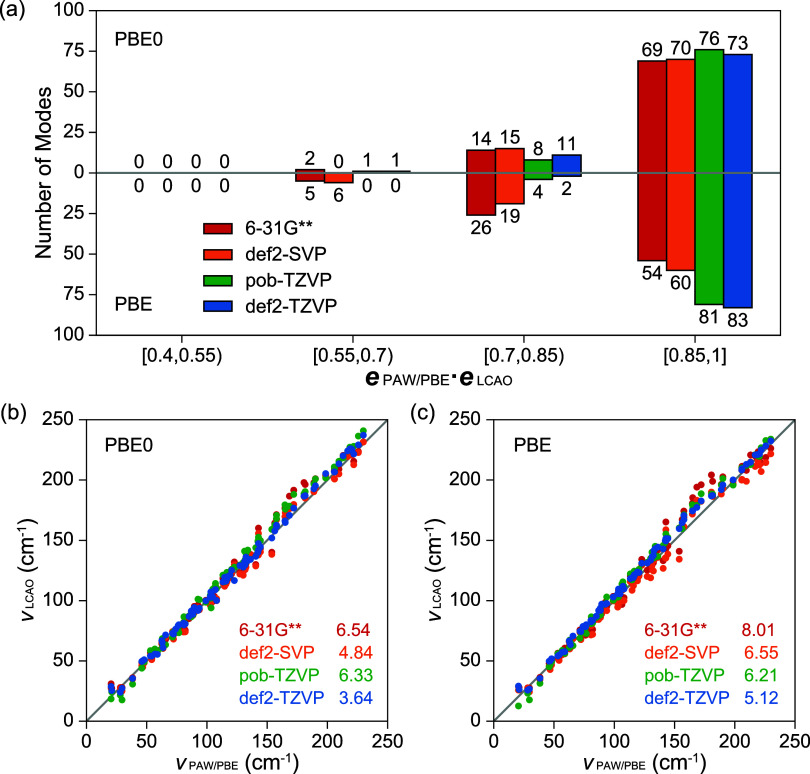
Similarities of intermolecular vibrational modes (0–250 cm^–1^) of form II paracetamol. (a) Distributions of dot products between mode eigenvectors obtained by PAW/PBE (***e***_PAW/PBE_) and those by LCAO/PBE­(PBE0) (***e***_LCAO_). (b, c) Phonon frequencies calculated by LCAO/PBE­(PBE0) (ν_LCAO_) as functions of phonon frequencies by PAW/PBE (ν_PAW/PBE_). Root-mean-square-deviations are annotated with legends.

Histograms of dot products suggest good similarities between PAW-based and LCAO-based vibrations, since no poor overlap (***e***_PAW/PBE_· ***e***_LCAO_ < 0.4) is identified in all cases. Similar to Raman spectra, def2-TZVP achieves the best agreement with PAW. The pob-TZVP basis set performs very differently between FI and FII, with FI phonons deteriorated due to the severely underestimated stacking height between the layered hydrogen bond networks, whereas its results for FII agree well with those from the larger def2-TZVP and PAW basis sets. A similar trend is also observed by comparing 6–31G** with def2-SVP, where, for FI, 6–31G** is close to the converged results and outperforms def2-SVP; but comparable results are achieved for FII. Although 6–31G** has fewer atomic orbitals than def2-SVP, its more diffusive valence orbitals better describe the interlayer dispersion interactions. This stresses that the diffusive outer atomic orbitals are critical to reduce the overbindings within molecular crystals, which originates from BSSE and becomes more prominent in FI due to the increased π–π stacking.

With def2-TZVP, the PBE0-based phonon eigenvectors exhibit lower similarities than those based on PBE, which should be attributed to the reference adopted (PAW/PBE). The difference is probably derived from the inconsistent description of strongly anharmonic modes, as previously analyzed. For double-ζ basis sets, regardless of the use of a different Hamiltonian, PBE0 contributes to a better alignment with the reference in FII, which is close to the results obtained with larger basis sets. However, this phenomenon is less prominent for FI where vibrations are more significantly influenced by the diffuseness of basis sets.

In addition to eigenvectors, good agreement in vibrational frequencies is illustrated between LCAO-based and PAW/PBE-based phonons below 250 cm^–1^, where the ascending order is preserved without significant deviation from the diagonal. The high similarity between the PBE0- and PBE-based frequencies clearly suggests that the Hamiltonian approximation exerts minimal influence on the frequencies of intermolecular vibrations. In comparison, the choice of basis sets has a considerable impact. Analogous to the trend observed for phonon eigenvectors, triple-ζ basis sets generally outperform double-ζ basis sets, with the def2-TZVP shows the best consistency with PAW/PBE.

### Intramolecular Vibrations and Anharmonicity

Although, as mentioned in previous studies,^7,11,13^ it is the low-frequency intermolecular vibrations that determine the relative stability of molecular crystal polymorphs, the calculated Raman spectra exhibit systematic deviations from experiments at higher frequencies (Figures S4 and S5), where intramolecular bending and stretching modes dominate. Such discrepancies originate from inherent errors in the harmonic approximation (HA) and the DFT energy surfaces, which indicates that, to reproduce the experimental spectra at higher frequencies, empirical corrections are inevitable regardless of the established benchmarks for basis set and Hamiltonian. It is interesting to examine whether such deviation can be simply accounted for by a uniform frequency scaling factor, as has been widely adopted for isolated molecules^62–64^ and, more recently, for molecular crystals.^48,65^ The published factors are applied to uniformly scale frequencies beyond 250 cm^–1^, since these factors are benchmarked against molecular references only. The values adopted are summarized in Table 2. No scaling factor has been reported for pob-TZVP/PBE as far as the authors are aware, where the same scaling factor as def2-TZVP/PBE is adopted considering the similar phonon frequencies reported by both methods at higher frequencies. The PAW/PBE scaling factor is not available for the same reason. Given the overall agreement of Raman spectra it achieves below 2800 cm^–1^, 1.0 (i.e., no scaling) is adopted.

**Table 2:** Frequency Scaling Factors

	6–31G**	def2-SVP	pob-TZVP	def2-TZVP	PAW
PBE0	0.9509^a^	0.9555^a^	0.9594^b^	0.9591^a^	
PBE	0.9848^a^	0.9904^a^	0.9923^c^	0.9923^a^	1.0

a See ref 64.

b See ref 48.

c The scaling factor of def2-TZVP/PBE-D3­(BJ) in ref 64 is used.

The high-frequency O–H and N–H stretching modes yield disproportionately large deviations, where distinct frequencies are reported for nearly identical vibrations, making the uniform frequency scalings to fail (Figures S7 and S9). In addition, the reference frequencies by PAW/PBE also significantly deviate from the experiments, as revealed by Raman spectra beyond 2800 cm^–1^ (Figures S4 and S5). This might be explained by the non-negligible nuclear quantum effects of the delocalized hydrogen atoms within X–H bonds, where X is a strongly electronegative element. To evaluate the influences of nuclear quantum effects, anharmonic frequencies of O–H and N–H stretching modes are calculated and compared with their harmonic counterparts in Table S4. Analysis in the Supporting Information suggests a better agreement obtained by PBE0, while a higher level of theory is required for an optimal alignment with experimental spectra. Nevertheless, both harmonic and anharmonic frequencies of the studied modes exhibit proximity between FI and FII, since these modes are highly intramolecular and dominated by the covalent X–H bonds. Therefore, prominent error cancellations are expected for the energy rankings of polymorphs, preserving the reliability of HA to study the relative stability of polymorphs.^12^

### Relative Stability

Thermal effects are non-negligible for quantitative evaluations of the relative stability between FI and FII owing to their subtle energy differences. Though intermolecular vibrations at low frequencies are widely known to be critical sources of lattice entropy, their influences on the relative stability of paracetamol remain unclear. In Figure 5, contributions from various terms of Gibbs free energy (eq 3) are partitioned to illustrate their roles in stabilizing FI over FII under 0 K, 0 MPa and 298 K, 0 MPa. Frequencies are scaled by the factors listed in Table 2.

**Figure 5:**
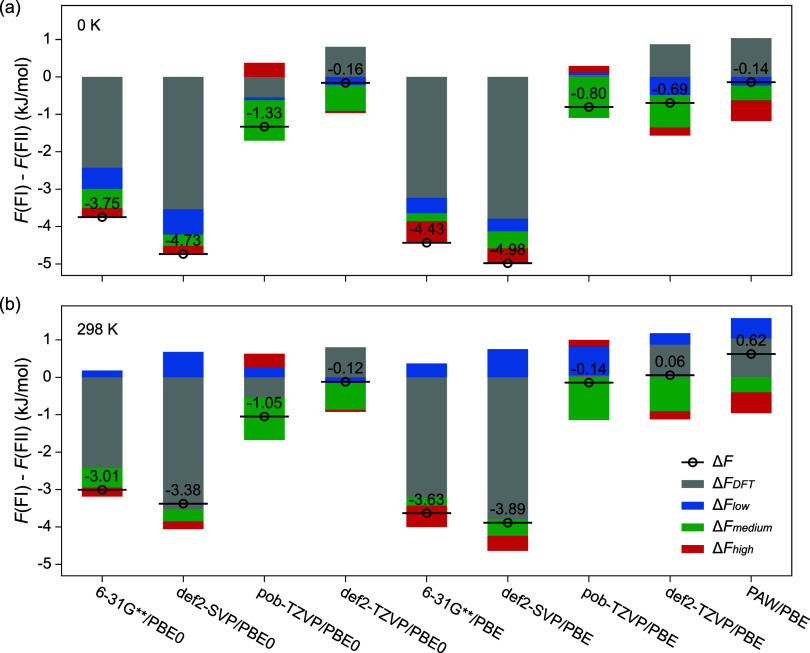
Contributions of DFT-based electronic energy (Δ*F*_DFT_), intermolecular vibrations below 250 cm^–1^ (Δ*F*_low_), intramolecular vibrations between 250 and 1750 cm^–1^ (Δ*F*_medium_), and hydrogen stretchings beyond 2500 cm^–1^ (Δ*F*_high_) to the Gibbs free energy difference between form I and form II paracetamol (Δ*F*) at (a) 0 K and (b) 298 K and zero pressure. Energies are reported in kJ/mol per formula (C_8_H_9_NO_2_).

The relative stability at 0 K is correctly captured in all cases, with Δ*F* within the range reported in the literature.^12,32^ However, Δ*F*_DFT_ shows that as the diffuseness of basis set increases, FII, rather than FI in experiments, becomes the energetically favorable phase. Including Fock exchange stabilizes FI by less than 1 kJ/mol, suggesting that basis set approximation dominants Δ*F*_DFT_. This phenomenon might originate from the overestimated *V*_DFT_ due to BSSE, which is more notable with double-ζ basis sets and FII (Table 1). The expanded lattice of FII reduces its binding energy and makes FI more favorable, coinciding with the reality. As the size of basis sets converges, this phenomenon becomes less prominent, making FII increasingly favorable. Therefore, the importance of including thermal effects in polymorph energy rankings is clearly demonstrated, as the differences in zero-point energy counterbalance those in electronic energy and stabilize FI at 0 K, especially when large basis sets are adopted. Except for the case of pob-TZVP, where inconsistent geometries and vibrations are predicted, zero-point energies throughout the frequency range stabilize FI over FII, as indicated by the negative Δ*F*_low_, Δ*F*_medium_, and Δ*F*_high_.

Influences of entropy manifest at finite temperatures, as illustrated in Figure 5b. The largest shifts are observed for Δ*F*_low_ due to thermal activation of intermolecular vibrations, while Δ*F*_medium_ and Δ*F*_high_ remain unchanged, indicating that contributions from intramolecular vibrations are limited to zero-point energies. Thermal activations at room temperature lead to greater increases in entropy of FII, which can be explained by the higher rotation freedom of the methyl groups (Figure S1). This phenomenon reduces the free energy difference and is consistent with the experimental phase diagram.^28^ However, the reversed relative stability is reported by large def2-TZVP and PAW basis sets, making FII the thermodynamically favorable phase under ambient conditions. A large proportion of positive Δ*F* originates from electronic energy Δ*F*_DFT_, which remains constant at finite temperatures, since thermal expansions are neglected and geometries are optimized at the athermal limit. Experimentally, a larger expansion rate of FII is observed,^25,27^ probably due to the increased entropy. The more prominent decreases in the density of FII could reduce the intermolecular bindings and Δ*F*_DFT_, indicating also the importance of thermal expansions in reproducing the relative stability of molecular crystal polymorphs.

Counterintuitively, under ambient conditions, the relative stability between FI and FII is correctly captured with double-ζ basis sets, regardless of the large deviations in geometry and phonons from those measured. To further evaluate the relative stability obtained by various methods, in Figure 6, temperature–pressure (*T*–*p*) phase diagrams of FI and FII (0–400 K, 0–1000 MPa) are plotted with the experimentally constructed topological phase diagram.^28^ The def2-TZVP-based and PAW-based phase boundaries agree with the general trend of the reference. As the size of basis set convergences, the constant-volume assumption of HA becomes the major source of errors, where increased deviations are expected at higher temperatures or pressures. Therefore, neglecting volume effects leads to larger deviations in the phase boundary at low-*T*, high-*p* and high-*T*, low-*p* regions. Better agreements are observed within medium *T* and *p* regions around 100–200 K and 0–200 MPa. In comparison, despite the correct energy ranking, there are significant errors in phase boundaries obtained by double-ζ basis sets. This result supports the previous analysis, that energy rankings based on double-ζ basis sets might benefit from the overestimated lattice volume and the consequent negative Δ*F*_DFT_, rather than a reliable prediction of all the terms of Δ*F*. In addition, PBE0 reduces the gradient of phase boundaries, alleviating errors of the constant-volume assumption at higher temperatures. This is due to the slightly lower Δ*F*_low_ at finite temperatures (Figure 5), indicating reduced contributions of the entropic term that stabilizes FII over FI. It probably originates from the different descriptions of intermolecular vibrations with stronger anharmonicity, as previously discussed.

**Figure 6:**
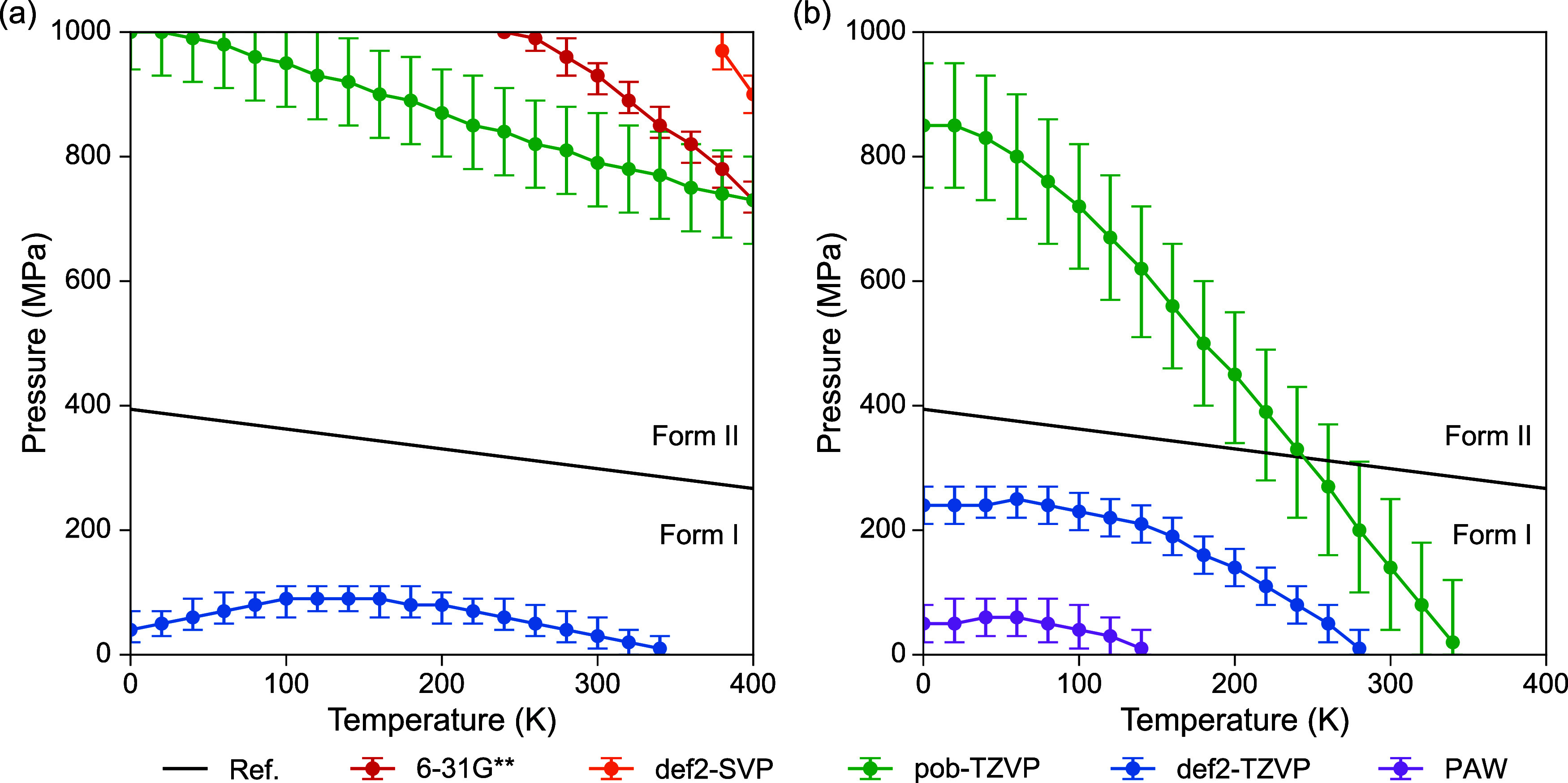
(a) PBE0- and (b) PBE-based temperature–pressure (*T*–*p*) phase diagrams of form I and form II paracetamol with *T* ranging from 0 to 400 K and *p* ranging from 0 to 1000 MPa. Solid black lines denote the topological *T*–*p* phase diagram reported by Espeau et al.,^28^ as *p*(MPa) = −0.3182*T* (K) + 394.25. Free energy differences less than ±0.1 kJ/mol are denoted by error bars.

## Conclusions

As critical parameters underpinning DFT simulations, the influences of various basis sets and Hamiltonians on the geometry, lattice vibration, and relative stability of paracetamol polymorphs, namely form I and form II, are evaluated, with the emphasis on low-frequency intermolecular vibrations below 250 cm^–1^. These properties are analyzed in detail as they are closely related to HA and Gibbs free energy, which are, respectively, the method and the criterion commonly adopted for polymorph energy rankings. A coherent convergence to the nearly complete plane-wave basis sets has been demonstrated, suggesting the dominant role of basis sets in controlling the precision of lattice dynamics. In particular, consistency with reference to the plane-wave achieved by the def2-TZVP basis set offers valuable insight into the best affordable precision of Gaussian basis sets in practical CSP workflows. Comparisons with published experimental measurements suggest that the vibrational properties predicted by double-ζ basis sets largely benefit from fortuitous error cancellations, whose transferability is questionable. The different converging trends with respect to theoretical and experimental references clearly distinguish the competing source of errors, and learning this is essential for predictive modeling of material thermodynamics.

The current work reveals that the GGA-PBE Hamiltonian is sufficient for structural and vibrational properties of the prototype molecular crystal, paracetamol, when used with large basis sets, and incorporating Fock exchange yields marginal improvements only. This finding might be applicable to other global hybrid functionals mixed with fixed percentages of Fock exchange when compared to their GGA counterparts, since the HF Hamiltonian is identical. However, their DFT Hamiltonians might largely differ from each other and need further benchmarking. Contributions from Fock exchange are more prominent with double-ζ basis sets, but it is so far unclear whether this conclusion is transferable to other molecular crystals or density functionals. Nevertheless, Fock exchange has been proven critical for the reliable estimation of other properties, such as magnetism and nonlinear optics,^66–68^ and this study has suggested a practical strategy to this end: the hybrid functional calculations based on def2-TZVP are expected to be comparable to the plane-wave-based hybrid functional calculations, which usually require prohibitive computational costs for molecular crystals.

The subtle energy difference in polymorph ranking puts forward stricter requirements in the precision of lattice dynamics, since both static and vibrational energies are involved. The zero-point energy is found to play a critical role in stabilizing FI over FII, whereas the higher entropy in FII drives the phase transition at high temperatures. At lower levels of precision, such as double-ζ basis sets, these delicate contributions are obscured by errors in DFT static energy, leading to the “artificially correct” relative stability that is not reproducible in *T*–*p* phase diagrams. In addition, errors of the neglected thermal expansion increasingly manifests at higher *T* or *p*, indicating the failure of the constant-volume assumption and, consequently, the HA-based thermodynamics in these systems. Within the framework of lattice dynamics, this can only be addressed with the quasi-harmonic approximation, which will be demonstrated in detail in an upcoming paper.

This study benchmarks the numerical precision of multiple basis sets and Hamiltonians from a practical perspective, which reveals probably the best affordable precision when ranking the free energies of molecular crystal polymorphs, considering the computational cost listed in Table S5. Nevertheless, from a purely theoretical point of view, using the plane-wave basis set rather than extrapolating to the complete basis set limit seems to be a compromise, even though a coherent convergence is obtained. Based on the conclusions of this study, we believe that such extrapolation can be performed confidently with DFT Hamiltonian, where calculations of Fock exchange might be unaffordable for systems with comparable sizes. In this regard, conclusions drawn from this work also provide references for future research dedicated to converging theoretical methods.

To summarize, a benchmarking of basis set and Hamiltonian has been conducted to identify the predominant factor influencing the geometrical and vibrational properties derived from DFT simulations. Such properties are distinctly relevant, and usually decisive, to the energy rankings of molecular crystal polymorphs, which remain challenging to pharmaceutical development and materials discovery based on *ab inito* methods. Therefore, the benchmarking tests presented here provide a basis for the reasonable choices of basis set and Hamiltonian to promote further investigations regarding the polymorphism of molecular crystals.

## Supplementary Material



## Notes

Further details on computational setups; additional results and discussion on Raman spectra and anharmonicity; the sorted phonon frequencies by all the benchmarked methods (PDF)
