# Supplementary material for: Influences of Basis Set and Electronic Exchange-Correlation on Low-Frequency Vibrations and Stability of Paracetamol Polymorphs
Source: J Chem Theory Comput. 2025 Oct 1;21(19):9832–43. doi: 10.1021/acs.jctc.5c00599 (PMC12529917; doi:10.1021/acs.jctc.5c00599)
Supplement: Supplementary file 1 [file ct5c00599_si_001.pdf]

Supporting Information: Influences of basis set and electronic exchange-correlation on low-frequency vibrations and stability of paracetamol polymorphs

Huanyu Zhou,* Giuseppe Mallia, and Nicholas M. Harrison

Department of Chemistry, Imperial College London, White City Campus, 80 Wood Lane, London, W12 0BZ, United Kingdom

E-mail: huanyu.zhou20@imperial.ac.uk

S1 Computational details

The computational parameters of the linear combination of atomic orbitals (LCAO) and the projected augmented wave (PAW) methods as implemented respectively in CRYSTAL23[1,2] (C23) and Quantum Espresso 7.3.1[3,4] (QE) are summarized in this section.

S1.1 Self-consistent field (SCF)

For both C23 and QE calculations, the total energy is converged to 10^{-9} Hartree per cell. Important keywords in the SCF block used by C23 are listed below:

```
DFTD3
VERSION
4
ABC
```

```
END
GCPAUTO
TOLDEE
9
FMIXING
50
TOLINTEG
8 8 8 8 24
SHRINK
0 4
4 4 2
ENDSCF
```

For def2-TZVP, to address the quasi-linear dependence of large basis sets, the following keywords are used:

```
LDREMO
10
TOLINTEG
8 8 8 8 30
```

Keywords used in the `SYSTEM`, `ELECTRONS`, `ATOMIC_SPECIES` and `K_POINTS` blocks of QE are listed below:

```
&SYSTEM
  ecutwfc          = 100
  ecutrho          = 400
  occupations      = 'fixed'
  input_dft        = 'pbe'
  vdw_corr         = 'dft-d3'
  dftd3_version    = 4
  dftd3_threebody = .TRUE.
/
&ELECTRONS
  conv_thr         = 2.0D-9
/
ATOMIC_SPECIES
  O  16.00  O.pbe-n-kjpaw_psl.1.0.0.UPF
  N  14.01  N.pbe-n-kjpaw_psl.1.0.0.UPF
  C  12.01  C.pbe-n-kjpaw_psl.1.0.0.UPF
  H   1.008 H.pbe-kjpaw_psl.1.0.0.UPF
K_POINTS {automatic}
  4 4 2 0 0 0
```

PAW pseudopotentials from the PSlibrary[5] are used.

S1.2 Geometry optimization

To avoid imaginary frequencies in phonon calculations, geometries of form I and II paracetamol crystals are optimized with slightly different criteria between C23 and QE due to the different options available. Keywords used in the `OPTGEOM` block of C23 are listed below:

```
OPTGEOM
TOLDEG
0.00010
TOLDEX
0.00040
ENDOPT
```

Keywords of the `CONTROL`, `IONS` and `CELL` blocks used by QE are:

```
&CONTROL
  calculation   = 'vc-relax'
  etot_conv_thr = 1.0D-5
  forc_conv_thr = 1.0D-4
  tprnfor       = .true.
  tstress       = .true.
/
&IONS
  ion_dynamics  = 'bfgs'
  upscale       = 100
/
&CELL
  cell_dynamics = 'bfgs'
  cell_dofree   = 'ibrav'
/
```

For C23 and QE calculations, the lattice structures are relaxed with the BFGS method, and the energy gradients are not greater than $1{\times}10^{-4}$ Hartree.Bohr^{-1}. Symmetry is kept throughout the optimization, while the cell parameters and atomic coordinates are fully relaxed.

S1.3 Lattice dynamics

The finite displacement method is adopted with the default displacement lengths of C23 and PHONOPY, respectively. For Gaussian basis set calculations based on C23, the displacement is 0.003 Å, while for plane-wave calculations based on QE and PHONOPY, the displacement

is 0.02 Bohr. The displacement of PAW/PBE calculation on FII is increased to 0.06 Bohr. The difference originates from the inconsistent truncation criteria of Coulomb and exchange series, which are fixed for lattice dynamics as implemented in C23,[2] whereas PHONOPY, as an external package, requires larger displacements to reduce the numerical noises on the potential energy surface (PES). Besides, in PAW calculations, only marginal influences are induced by inconsistently displaced internal coordinates, since the plane waves are defined with respect to the periodic lattice. All the reported displacements are considered to impose negligible influences on phonon modes, as they are within the range of the published lattice dynamic workflow,[6] and the calculated vibrational modes show good consistency.

S1.4 Anharmonicity

Geometries optimized by C23 are displaced along the eigenvectors of selected vibrational modes by the `SCANMODE` keyword. The numerically computed total energies of the displaced geometries are compared with their analytically obtained counterparts based on harmonic approximations to characterize the influences of anharmonicity.

Anharmonic frequencies of O−H and N−H stretching modes are calculated with the `ANHARM` keyword of C23, which solves the one-dimensional nuclear Schrödinger equation by numerically scanning the specified X−H stretching mode.[7]

S2 Supplementary results

S2.1 Candidate basis sets

The atomic basis sets benchmarked in this study are shortlisted from the candidates detailed in **Table S1**, which also includes the commonly adopted Dunning correlation-consistent 'cc' basis sets[8] and the augmented Dunning 'aug-cc' basis sets.[9] The basis sets studied in the manuscript are marked in bold.

Table S1: The minimum eigenvalue of overlap matrices and the availability of gCP correction

**Basis Set**	$\boldsymbol{\lambda_1}$	Available for gCP
**6-31G****	$\mathbf{2.74\times10^{-4}}$	**Yes**
**def2-SVP**	$\mathbf{1.43\times10^{-4}}$	**Yes**
**pob-TZVP**	$\mathbf{6.53\times10^{-4}}$	**Yes**
**def2-TZVP**	$\mathbf{8.01\times10^{-7}}$	**Yes**
def2-TZVPP	5.70×10^{-7}	No
def2-TZVPD	-6.03×10^{-6}	No
def2-QZVP	-4.75×10^{-7}	No
cc-PVDZ	1.70×10^{-4}	Yes
cc-PVTZ	6.10×10^{-6}	No
cc-PVQZ	1.71×10^{-7}	No
aug-cc-PVDZ	-3.89×10^{-6}	Yes
aug-cc-PVTZ	-7.77×10^{-6}	No

Compared with isolated systems, the numerical instability arising from the basis set (quasi-)linear dependence is particularly pronounced with periodic crystals due to their densely packed geometry.[10] Hence, the first criterion of shortlisting is the minimum eigenvalue λ_1 of overlap matrices over the 4×4×2 Monkhorst-Pack grid of form I (FI) and form II (FII) paracetamol crystals. The negative or nearly-zero λ_1 significantly influences the numerical stability when obtaining the inverse of overlap matrices with the standard Cholesky decomposition scheme implemented in SCF iterations.[11] This scheme directly collapses with the negative λ_1 of the def2-TZVPD, def2-QZVP and 'aug-cc' basis sets, making any further investigation infeasible. The λ_1 of def2-TZVP is less than 10^{-6}, indicating that twelve decimal places out of sixteen of a double-precision float become redundant during decomposition, which explains the higher threshold adopted in **Section S1.1**.

The availability of the geometrical counterpoise (gCP) method is another important consideration, so the same correction method for the basis set superposition error (BSSE) is adopted for all calculations, ensuring consistent and directly comparable results. Having gCP also facilitates the comparison with experiments, where the alignment to references involves correcting the BSSE-influenced static internal energy. These complexities are beyond the current scope but, fortunately, can be approximated in a general and simple way with gCP,

making it possible to focus the analysis on vibrations. Therefore, the Dunning 'cc' series are not benchmarked, since only the cc-PVDZ basis set is parameterized.[12] The Karlsruhe 'def2' basis sets up to def2-TZVP are systematically parameterized for gCP and exhibit good numerical stability, therefore they are shortlisted to illustrate the convergence of basis sets.

Based on the findings of this work, further extrapolation to the complete basis set limit might be viable with Dunning 'cc' basis sets, since they are numerically more stable at the quadruple-ζ level, where influences of BSSE become negligible for periodic systems even without gCP.[13] The less expansive GGA-PBE can be adopted, as this work has revealed the marginal influences of Fock exchange when used with larger basis sets. The 'cc' basis sets are also useful when the correlation consistency needs to be considered, for example, for optical properties or dispersion corrections. Nevertheless, the 'def2' basis sets are sufficient in this study based on electron ground states.

S2.2 Experimental and optimized lattice parameters

The experimental lattice parameters of FI and FII are obtained from published data, which are summarized in **Table S2**. The structure of FI is measured by Wilson[14] with neutron diffraction at 20K, which is available as HXACAN13 at the Cambridge Crystallographic Data Center (CCDC). The structure of FII is measured by Druzhbin *et al.*[15] with X-ray diffraction at 20K, which is available as HXACAN37 at CCDC. Both structures are reoriented according to the space group symmetry listed in **Table S2**.

The optimized lattice parameters of FI and FII are summarized in **Table S3**.

Table S2: Experimental lattice parameters of FI and FII[a]

**Polymorph**	**Space Group**	***a*** **(Å)**	***b*** **(Å)**	***c*** **(Å)**	***β*** **(deg)**
form I	$P1\ 2_1/c\ 1$	7.073(3)	9.166(3)	12.667(4)	115.51(2)
form II	$P2_1/b\ 2_1/c\ 2_1/a$	11.7552(3)	7.13941(11)	17.1714(2)	

[a] The Numbers standard uncertainties of the final digits are reported in parentheses.

Table S3: Optimized lattice parameters of FI and FII [a]

**Polymorph**	**Hamiltonian**	**Basis Set**	***a*** **(Å)**	***b*** **(Å)**	***c*** **(Å)**	***β*** **(deg)**
form I	PBE0	6-31G**	7.15 (1.15)	9.46 (3.19)	12.76 (0.76)	115.01 (-0.43)
		def2-SVP	7.20 (1.73)	9.45 (3.05)	12.92 (2.01)	115.32 (-0.16)
		pob-TZVP	7.15 (1.05)	8.69 (-5.20)	12.93 (2.10)	113.66 (-1.60)
		def2-TZVP	7.08 (0.17)	9.24 (0.75)	12.80 (1.02)	115.97 (0.40)
	PBE	6-31G**	7.12 (0.60)	9.46 (3.16)	12.80 (1.09)	113.73 (-1.55)
		def2-SVP	7.24 (2.42)	9.50 (3.62)	13.05 (3.05)	114.46 (-0.91)
		pob-TZVP	7.11 (0.51)	8.69 (-5.16)	13.02 (2.82)	113.37 (-1.85)
		def2-TZVP	7.04 (-0.43)	9.06 (-1.17)	12.86 (1.53)	114.40 (-0.96)
		PAW	7.01 (-0.86)	9.17 (0.09)	12.83 (1.30)	114.44 (-0.93)
form II	PBE0	6-31G**	11.81 (0.50)	7.44 (4.28)	17.31 (0.81)	90
		def2-SVP	11.89 (1.13)	7.52 (5.34)	17.35 (1.06)	
		pob-TZVP	11.54 (-1.86)	7.32 (2.49)	17.22 (0.30)	
		def2-TZVP	11.70 (-0.48)	7.26 (1.68)	17.17 (-0.00)	
	PBE	6-31G**	11.78 (0.24)	7.49 (4.97)	17.44 (1.59)	
		def2-SVP	11.96 (1.73)	7.62 (6.70)	17.60 (2.49)	
		pob-TZVP	11.53 (-1.88)	7.33 (2.67)	17.33 (0.92)	
		def2-TZVP	11.61 (-1.24)	7.27 (1.78)	17.26 (0.51)	
		PAW	11.62 (-1.16)	7.31 (2.37)	17.27 (0.60)	

[a] The percentage error respect to experimental data[14] are reported in parentheses.

S2.3 Low-frequency lattice vibrations

To characterize the anharmonicity of intermolecular modes, a numerical scan of their PES is performed by displacing the geometry with the corresponding eigenvectors. The def2-TZVP/PBE is adopted for numerical scans due to its agreement with experimental Raman spectra and relatively low computational costs. Two modes of FI and FII are visualized and scanned in **Figure S1**, where non-negligible differences are observed between Raman spectra based on def2-TZVP/PBE and def2-TZVP/PBE0. The rotation around molecular skeleton, *i.e.*, between the hydroxyl group and the methyl group, is observed in both FI and FII, while in FII, there is a coupling rotation of the methyl group. The difference between harmonic and anharmonic PESs illustrates the strong anharmonicity of both modes. The more significant anharmonicity of FII, as indicated by the larger deviation from harmonic PES, should be attributed to the extra rotational freedom of methyl groups.[16,17] The results suggest the potential influence of electron self-interaction errors, which become increasingly significant at the presence of strong inter-mode correlations, leading to the differences in Raman spectra. Besides, the inherent error in the sampling by Cartesian displacements might also be prominent, since rotation is involved in both modes. The rotational, or torsional, modes might be better described by internal redundant coordinates defined by molecular geometries, which, however, could increase computational loads and are rarely reported for periodic systems.

In comparison, modes associated with the highest Raman peak are visualized and scanned in **Figure S2**, where almost negligible anharmonicity is observed. Good consistency in peak position and intensity of Raman spectra is achieved between def2-TZVP/PBE and def2-TZVP/PBE0, which supports the analysis in the manuscript, that Hamiltonian imposes limited influences on low-frequency vibrational modes.

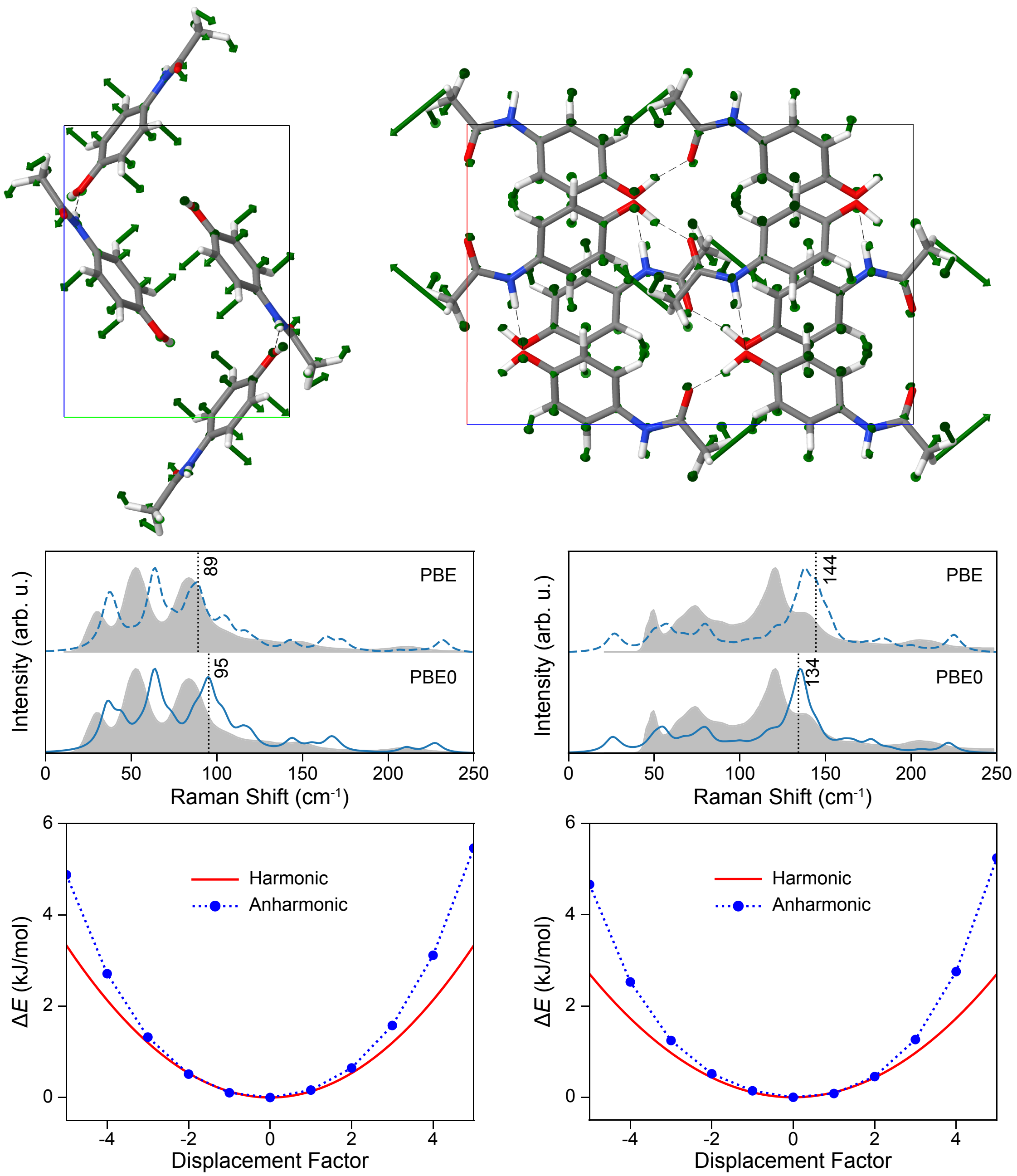

Figure S1: (left) The vibration, Raman spectra, harmonic and anharmonic potential energy surfaces (PES) of mode # 16 of FI (**Table S6**). (right) The vibration, Raman spectra, harmonic and anharmonic PESs of mode # 59 of FII (**Table S7**). Peak positions of corresponding modes are annotated with black dotted lines in Raman spectra. def2-TZVP/PBE is adopted to scan PESs. Experimental spectra adapted with permission from ref. 18. Copyright 2012 American Chemical Society.

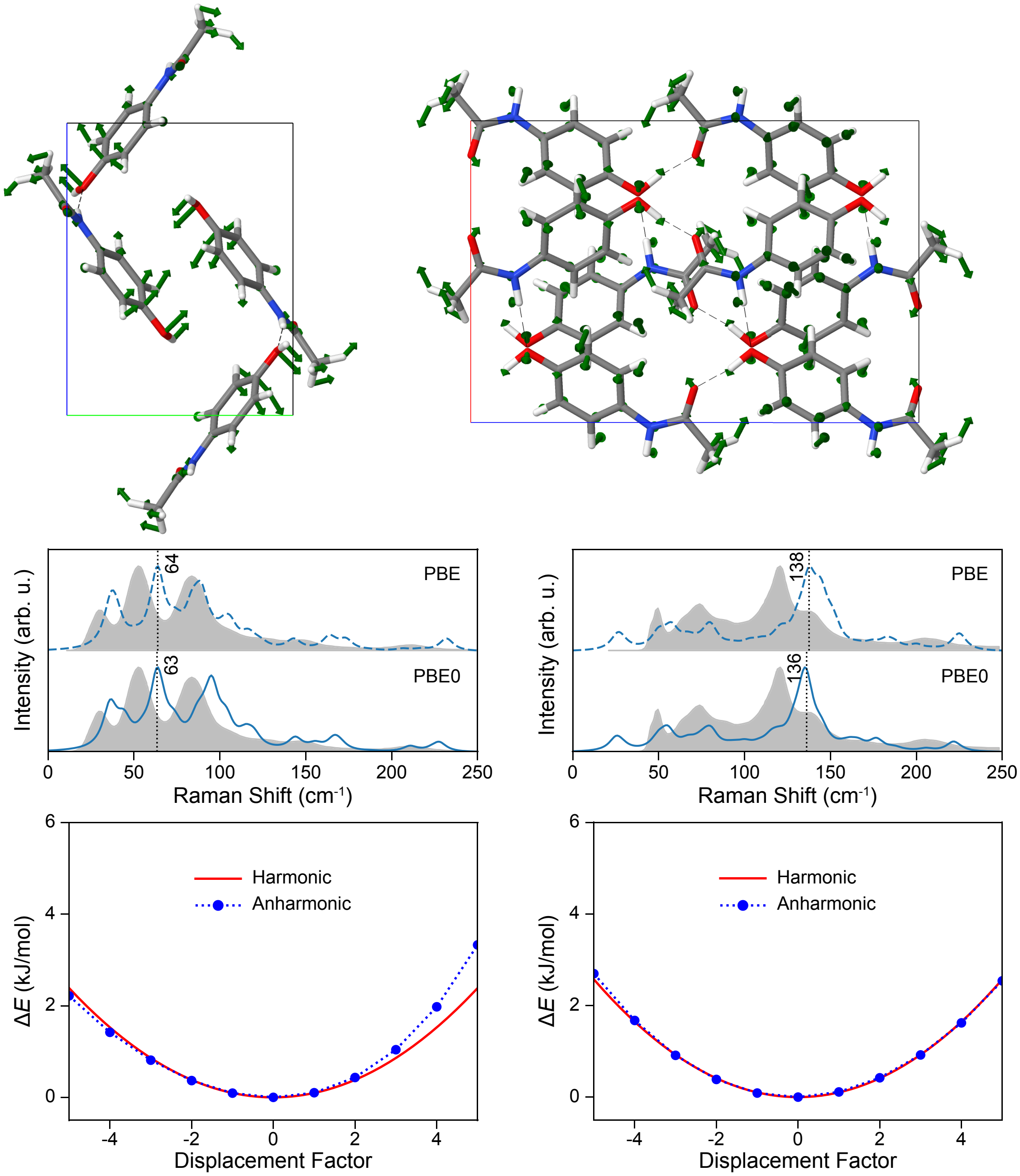

Figure S2: (left) The Raman spectra, harmonic and anharmonic PESs of mode # 10 of FI (**Table S6**). (right) The Raman spectra, harmonic and anharmonic PESs of mode # 55 of FII (**Table S7**). Peak positions of corresponding modes are annotated with black dotted lines in Raman spectra. def2-TZVP/PBE is adopted to scan PESs. Experimental spectra adapted with permission from ref. 18. Copyright 2012 American Chemical Society.

S2.4 Basis set superposition error

Significant inconsistencies in geometries and lattice vibrations have been predicted by the pob-TZVP basis set. Although, for FII, it achieves comparable results to those based on def2-TZVP and plane-wave, results of FI are severely deteriorated, which are outperformed by smaller double-ζ basis sets. Errors in the gCP,[19,20] which has been adopted throughout this study to correct the basis set superposition error (BSSE) of Gaussian orbitals, probably lead to these inconsistencies. In particular, as indicated by the equilibrium lattice parameters in **Table S3**, the error becomes increasingly prominent along the stacking direction of hydrogen-bonded layers (*i.e.*, b of **Figure 1**), where interactions are dominated by London dispersions. The delicate inter-layer bindings make this direction more vulnerable to numerical errors such as BSSE.

To evaluate the precision of gCP, the gCP-corrected binding energies E_{bind} are compared to counterpoise(CP)-corrected ones.[21] Unit cells of FI and FII are divided perpendicular to b into two layers to account for inter-layer BSSE, whose influences on geometry and vibrations are the most significant. The inter-layer distances are varied with fixed internal coordinates to investigate the dependence on the lattice parameter b. PBE is adopted for the results illustrated in **Figure S3** to reduce computational cost, since BSSE equivalently affects calculations with the same basis set; comparisons are made at the triple-ζ level, between pob-TZVP and def2-TZVP. The D3 correction term is subtracted from E_{bind} since its definition is ambiguous with the 'ghost atoms' required by CP correction.

At equilibrium distance and with the pob-TZVP basis set, a large energy difference of 12.30 kJ/mol per formula between gCP- and CP-corrected E_{bind} is observed for FI, while for FII the energy difference is 3.09 kJ/mol. This could be due to the distinct $\pi - \pi$ stacking configurations that contribute differently to BSSE. In FI, the benzene rings are parallel and overlapped between layers, which promotes $\pi - \pi$ stacking, while in FII the alignment of benzene rings is disrupted (**Figure 1**). A previous study reported larger errors in gCP for

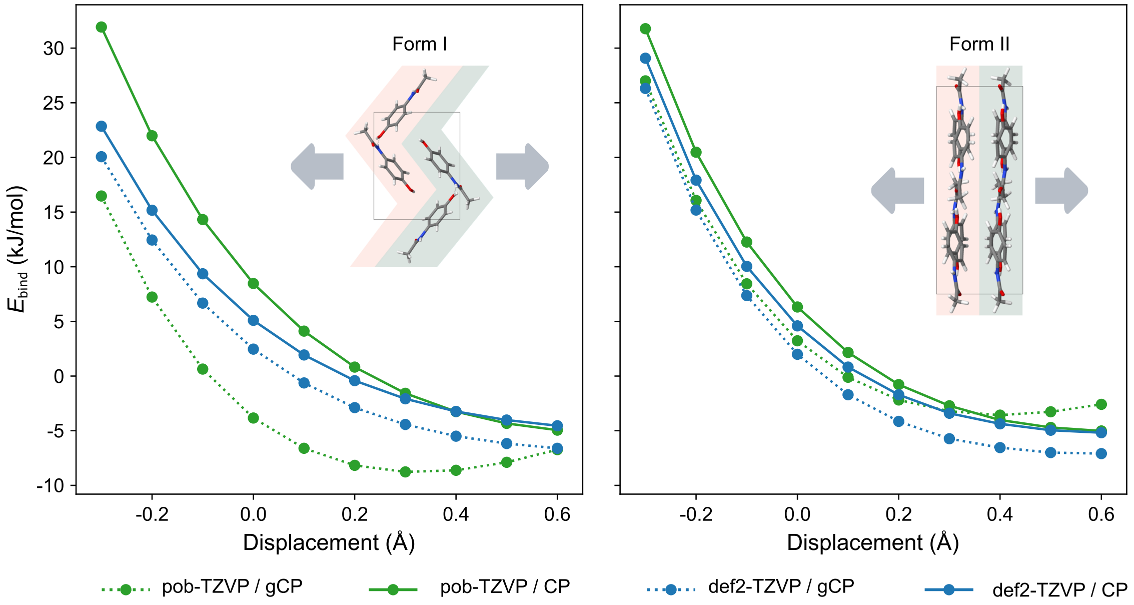

Figure S3: Interlayer binding energies E_{bind} of (left) FI and (right) FII corrected by the geometrical counterpoise (gCP) and the counterpoise (CP) methods. The partition of hydrogen-bonded layers and the directions of displacements are illustrated in insets.

$\pi - \pi$ stacked systems.[13] Besides, for both FI and FII, the asymptotics of gCP-corrected E_{bind} differ from those of CP-corrected E_{bind}, where an exponential decay is expected.[13,20] In comparison, errors in E_{bind} with def2-TZVP are no greater than 3 kJ/mol, with the asymptotics correctly reproduced. Therefore, the error of pob-TZVP probably originates from the combined effect of the geometry and gCP parameterization. The geometry of FI intensifies BSSE, while gCP fails to fully account for it. This finding is also indirectly characterized in **Figure 3bc**, where, except for a few outliers, pob-TZVP generally overestimates intermolecular phonon frequencies, suggesting the overbinded FI.

S2.5 Raman spectra and lattice vibrations beyond 250 cm^{-1}

Supplementary Raman spectra and lattice vibrations are presented in this section. In both the experimental and the simulated Raman spectra, discontinuities are observed around 240~320 cm^{-1} and around 1750~2940 cm^{-1}, which partition Raman spectra into three distinct regions characterized by different vibrational patterns:

- The low-frequency region, $\nu < 250$ cm^{-1}, as proposed in the manuscript, originates from the collective motions of molecules in lattice, and is dominated by the weak non-covalent interactions such as dispersion and hydrogen bonds.

- The medium-frequency region, 250 cm^{-1} $\leq \nu <$ 1750 cm^{-1}, originates from intramolecular vibrations, including all the molecular bending modes and the stretching modes of heavy atoms.

- The high-frequency region, $\nu \geq 2800$ cm^{-1}, originates from the stretching modes of X−H bonds, where X is an arbitrary element.

The medium- to high-frequency Raman spectra of FI and FII are illustrated in **Figure S4** and **Figure S5** respectively. The same experimental conditions as in the manuscript (λ=532 nm, T=300 K) are used. Similarities of intramolecular vibrational modes beyond 250 cm^{-1} are compared between LCAO- and PAW-based phonons, as illustrated in **Figure S6** to **Figure S9**.

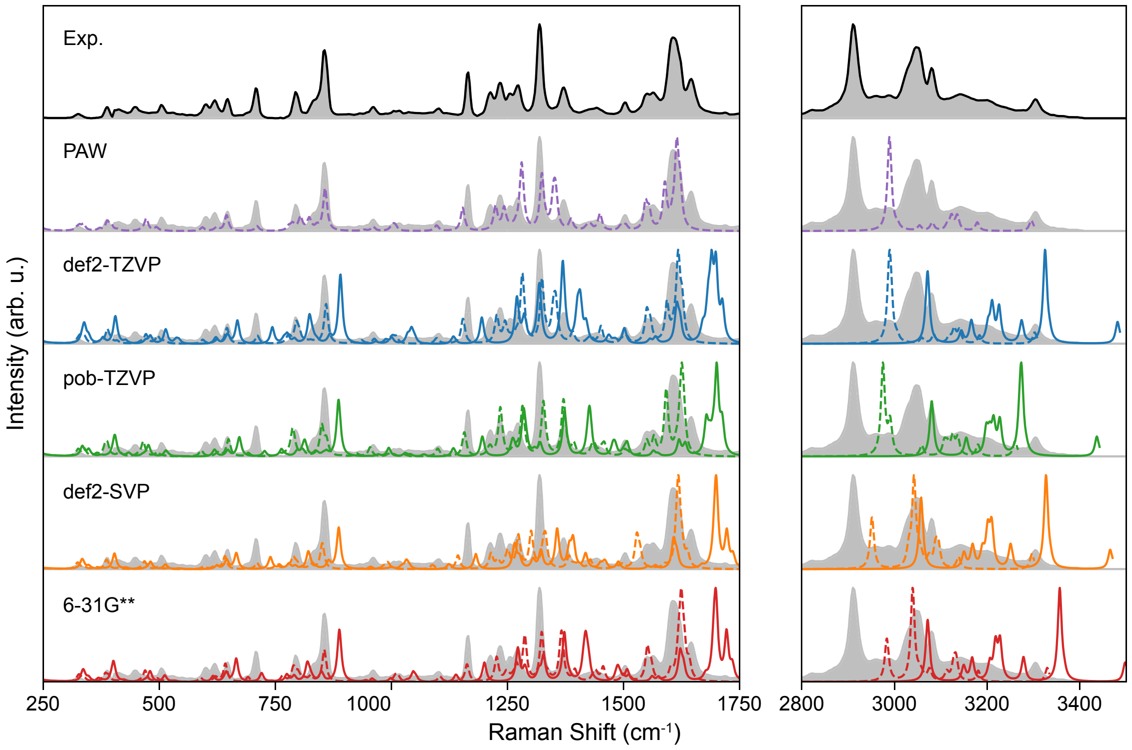

Figure S4: Calculated Raman spectra of FI (left) from 250 to 1750 cm^{-1} and (right) from 2800 to 3500 cm^{-1}. Spectra in solid lines are obtained by PBE0, while spectra in dashed lines are obtained by PBE. Intensities are normalized to the highest peak of each plot. Experimental spectra adapted with permission from ref. 18. Copyright 2012 American Chemical Society.

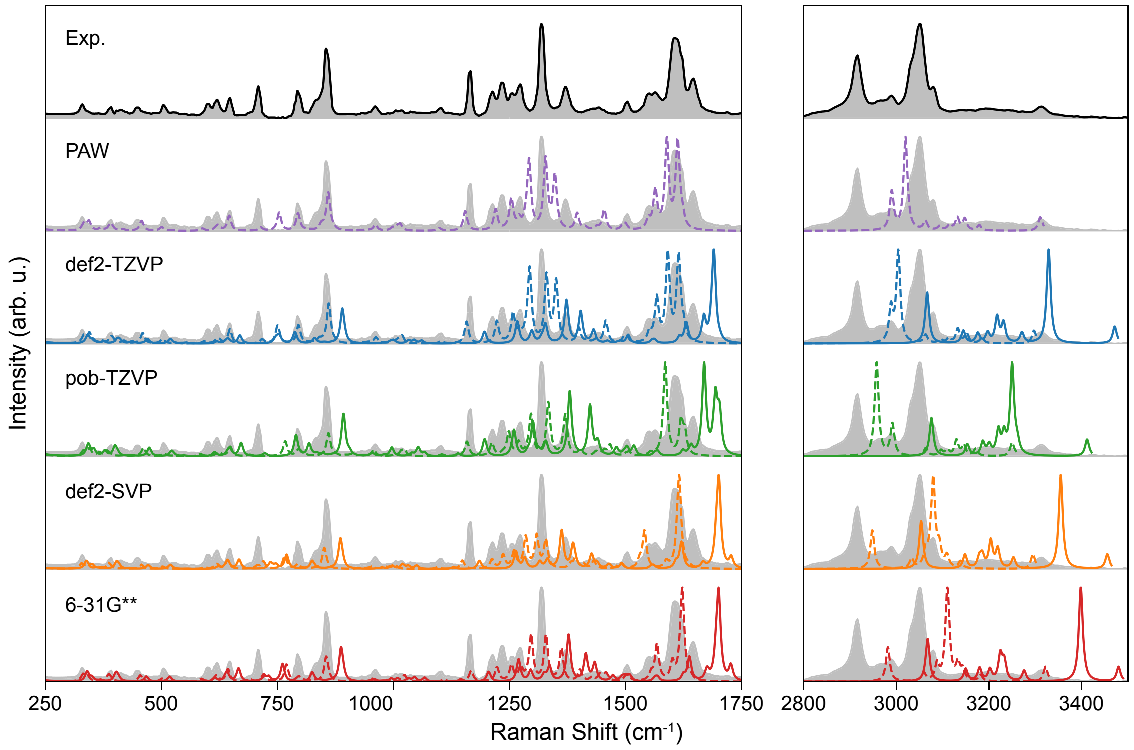

Figure S5: Calculated Raman spectra of FII (left) from 250 to 1750 cm^{-1} and (right) from 2800 to 3500 cm^{-1}. Spectra in solid lines are obtained by PBE0, while those in dashed lines are obtained by PBE. Intensities are normalized to the highest peak of each plot. Experimental spectra adapted with permission from ref. 18. Copyright 2012 American Chemical Society.

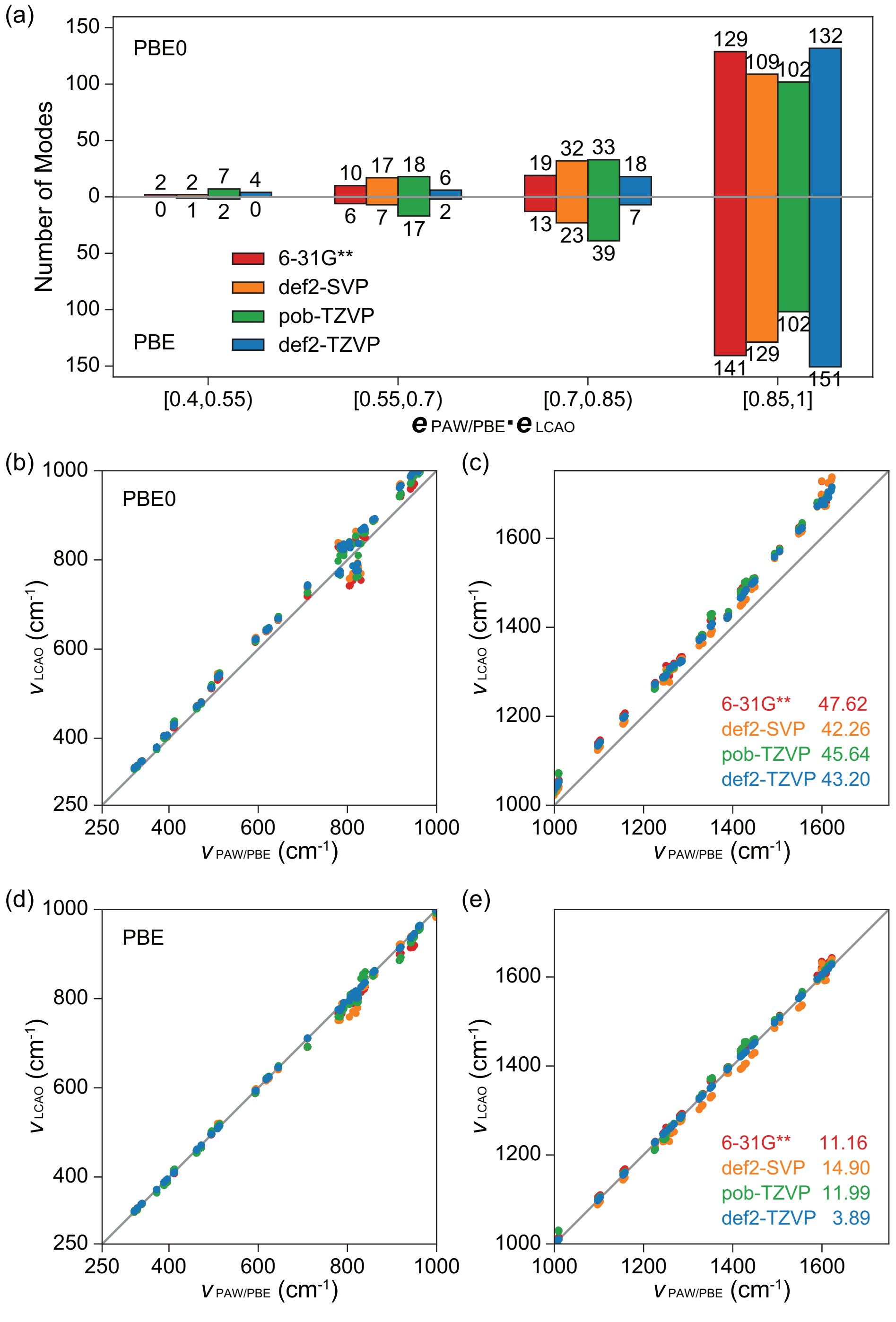

Figure S6: Similarities of medium-frequency vibrational modes (250∼1750 cm^{-1}) of FI. (a) Distributions of dot products between mode eigenvectors obtained by PAW/PBE ($\boldsymbol{e}_{\mathrm{PAW/PBE}}$) and those by LCAO/PBE(PBE0) ($\boldsymbol{e}_{\mathrm{LCAO}}$). (b)-(e) Phonon frequencies calculated by LCAO/PBE(PBE0) (ν_{LCAO}) as functions of phonon frequencies by PAW/PBE ($\nu_{\mathrm{PAW/PBE}}$). Figures are divided at 1000 cm^{-1} for visualization proposes. Root-mean-square-deviations are annotated with legends.

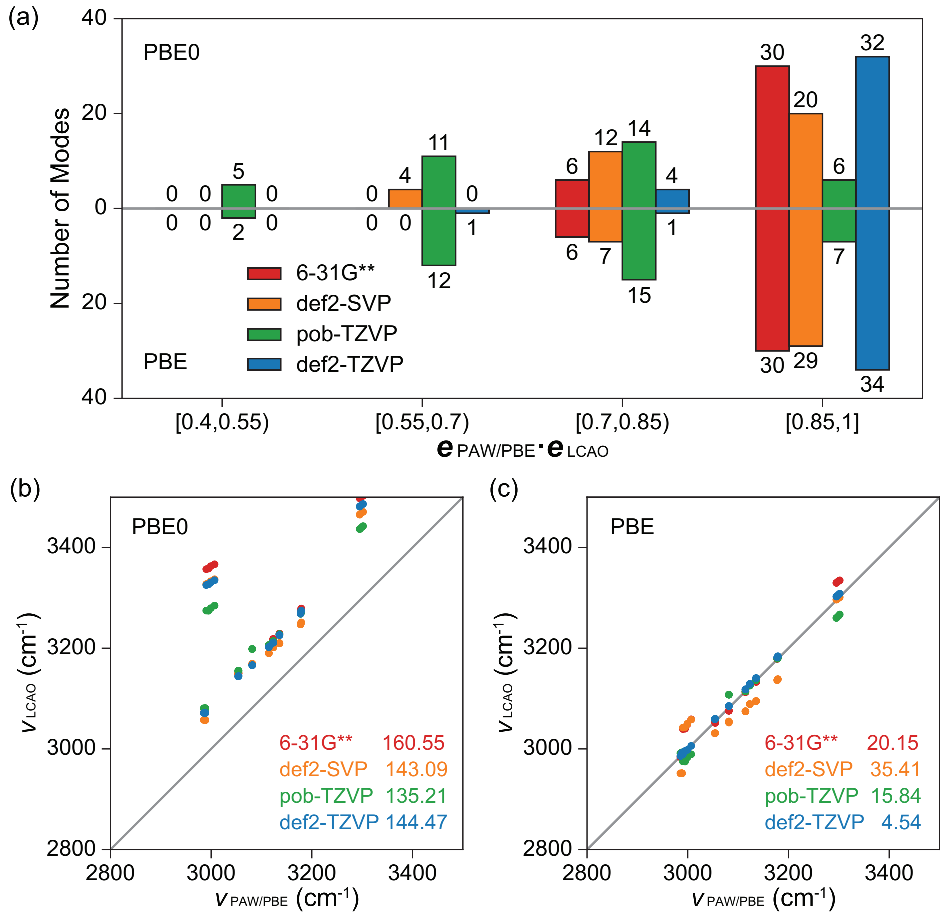

Figure S7: Similarities of high-frequency vibrational modes (≥2800 cm^{-1}) of FI. (a) Distributions of dot products between mode eigenvectors obtained by PAW/PBE ($\boldsymbol{e}_{\mathrm{PAW/PBE}}$) and those by LCAO/PBE(PBE0) ($\boldsymbol{e}_{\mathrm{LCAO}}$). (b)(c) Phonon frequencies calculated by LCAO/PBE(PBE0) (ν_{LCAO}) as functions of phonon frequencies by PAW/PBE ($\nu_{\mathrm{PAW/PBE}}$). Root-mean-square-deviations are annotated with legends.

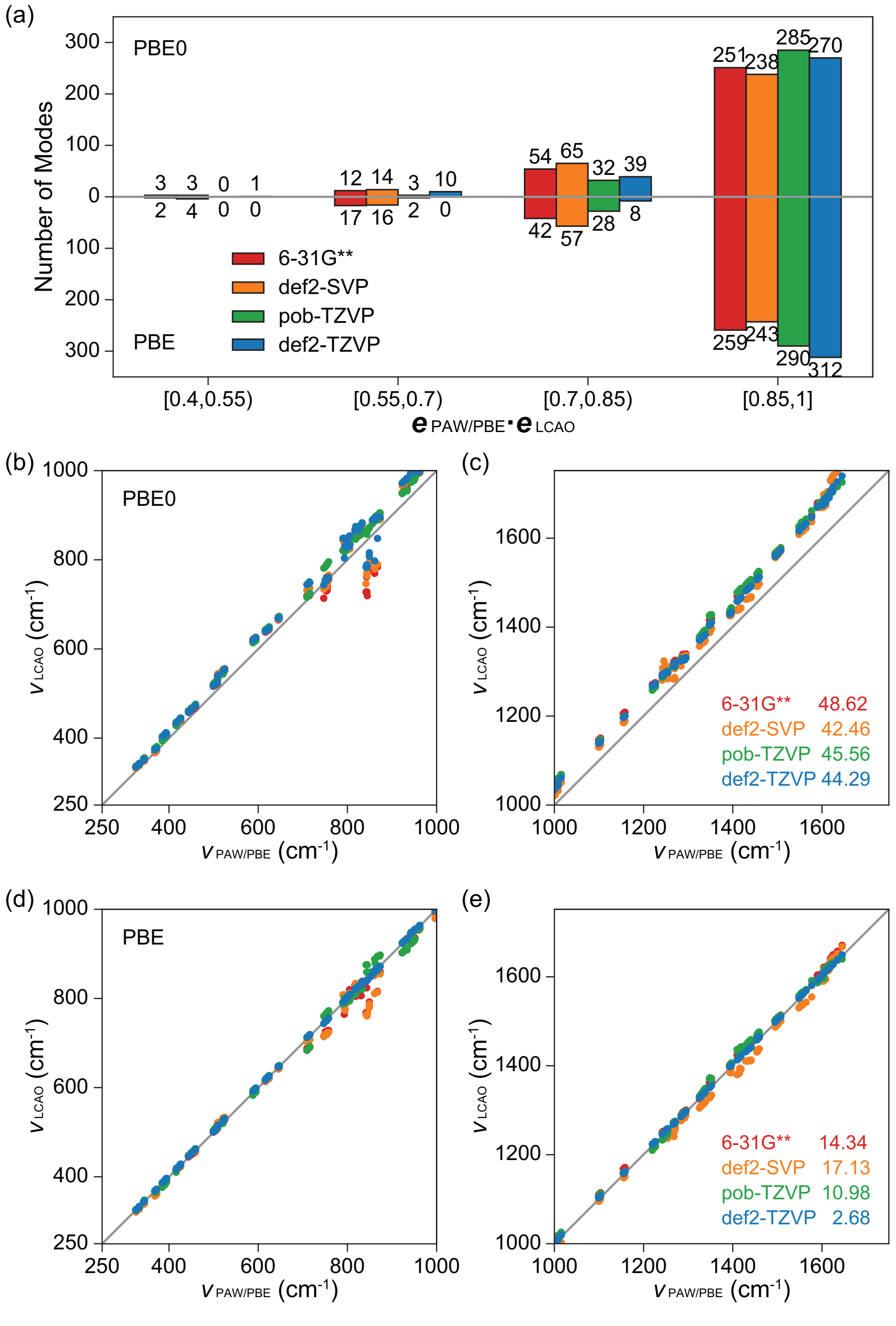

Figure S8: Similarities of medium-frequency vibrational modes (250∼1750 cm^{-1}) of FII. (a) Distributions of dot products between mode eigenvectors obtained by PAW/PBE ($\boldsymbol{e}_{\mathrm{PAW/PBE}}$) and those by LCAO/PBE(PBE0) ($\boldsymbol{e}_{\mathrm{LCAO}}$). (b)-(e) Phonon frequencies calculated by LCAO/PBE(PBE0) (ν_{LCAO}) as functions of phonon frequencies by PAW/PBE ($\nu_{\mathrm{PAW/PBE}}$). Figures are divided at 1000 cm^{-1} for visualization proposes. Root-mean-square-deviations are annotated with legends.

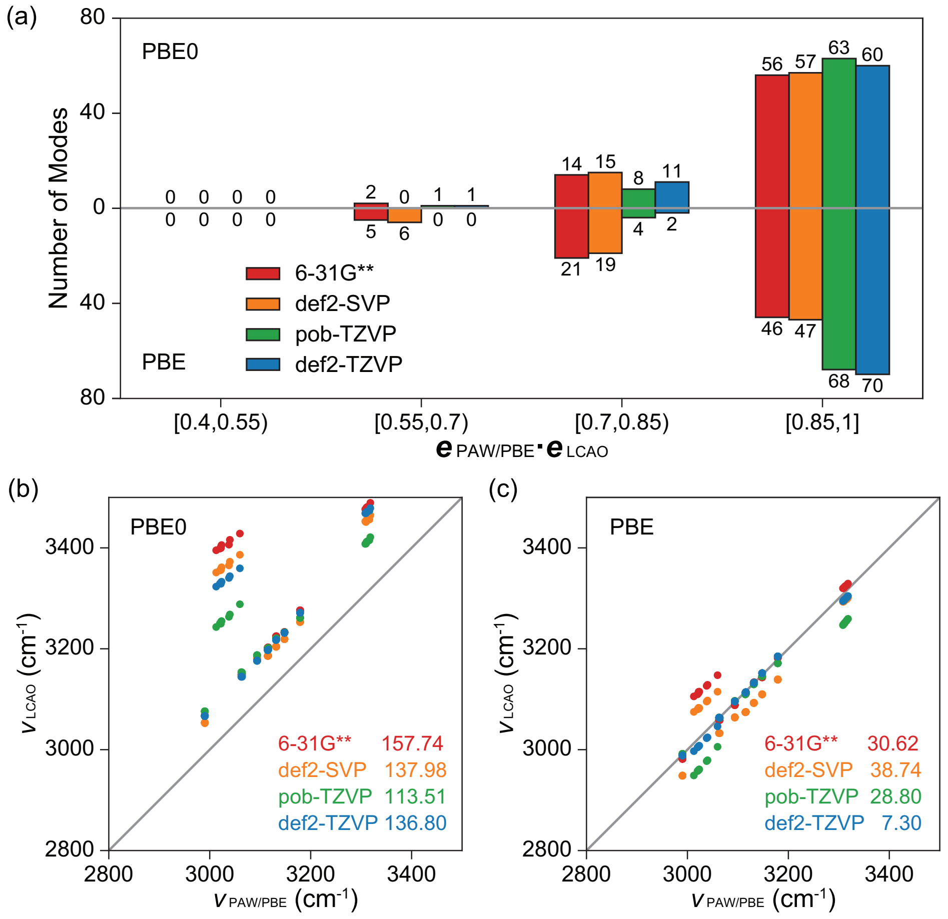

Figure S9: Similarities of high-frequency vibrational modes ($\geq$2800 cm^{-1}) of FII. (a) Distributions of dot products between mode eigenvectors obtained by PAW/PBE ($\boldsymbol{e}_{\mathrm{PAW/PBE}}$) and those by LCAO/PBE(PBE0) ($\boldsymbol{e}_{\mathrm{LCAO}}$). (b)(c) Phonon frequencies calculated by LCAO/PBE(PBE0) (ν_{LCAO}) as functions of phonon frequencies by PAW/PBE ($\nu_{\mathrm{PAW/PBE}}$). Root-mean-square-deviations are annotated with legends.

S2.6 Corrections of anharmonicity

Harmonic (ν^{ha}) and anharmonic (ν^{aha}) frequencies of the O−H and N−H stretching modes are reported in **Table S4**. Only LCAO-based results are reported due to technical restrictions. Nevertheless, anharmonic frequencies of PAW/PBE can be estimated based on those of def2-TZVP/PBE considering their similarities analyzed in the manuscript.

Table S4: Frequencies of O−H and N−H stretching modes. Unit: cm^{-1}

**Polymorph**	**Hamiltonian**	**Basis Set**	$\boldsymbol{\nu^{ha}}$**(OH)**	$\boldsymbol{\nu^{aha}}$**(OH)**	$\boldsymbol{\nu^{ha}}$**(NH)**	$\boldsymbol{\nu^{aha}}$**(NH)**
form I	PBE0	6-31G**	3357	3003	3498	3320
		def2-SVP	3327	2979	3466	3285
		pob-TZVP	3275	2895	3437	3235
		def2-TZVP	3312	2960	3470	3298
	PBE	6-31G**	3039	2600	3330	3135
		def2-SVP	3042	2643	3297	3108
		pob-TZVP	2975	2530	3260	3039
		def2-TZVP	2989	2548	3303	3094
form II	PBE0	6-31G**	3398	3076	3480	3293
		def2-SVP	3355	3033	3456	3270
		pob-TZVP	3250	2874	3412	3197
		def2-TZVP	3329	2989	3472	3283
	PBE	6-31G**	3110	2725	3322	3125
		def2-SVP	3080	2711	3295	3105
		pob-TZVP	2958	2533	3250	3022
		def2-TZVP	3004	2598	3297	3090

ν(OH) are generally red-shifted by over 300 cm^{-1} after the inclusion of anharmonicity. In comparison, ν(NH) are red-shifted by ∼200 cm^{-1}. The significant shift of frequency suggests the non-negligible anharmonicity of the high-frequency stretching modes of hydrogen, which becomes increasingly prominent at the delocalized hydrogen atom of hydrogen bonds. Furthermore, the anharmonicity of X−H bonds can probably act as an indicator of their binding strengths when various hydrogen bonds exist. The stronger anharmonicity of the O−H stretching mode suggests a more prominent hydrogen delocalization at the OH. . . O=C bond and, therefore, a stronger hydrogen bond. This supports previous studies where the relative strengths of OH. . . O=C and NH. . . OH of FI are indirectly characterized by the stiffness matrix.[22,23] Results of this work also agree with the findings by path-integral molecular

dynamics,[24] where delocalized hydrogen is the main contributor to anharmonicity due to the increased nuclear quantum effects. However, there are still large disagreements between the PBE0- and PBE-based frequencies, probably as the result of electron self-interactions.

The Raman spectra of FI and FII beyond 250 cm^{-1} with anharmonic corrections are illustrated in **Figure S10** and **Figure S11**, respectively. The LCAO-based harmonic frequencies of the O−H and N−H stretching modes are substituted by the anharmonic frequencies listed in **Table S4**, while frequencies based on PAW/PBE are kept. Frequencies of other modes are uniformly scaled by the empirical factors listed in **Table 2** of the manuscript.

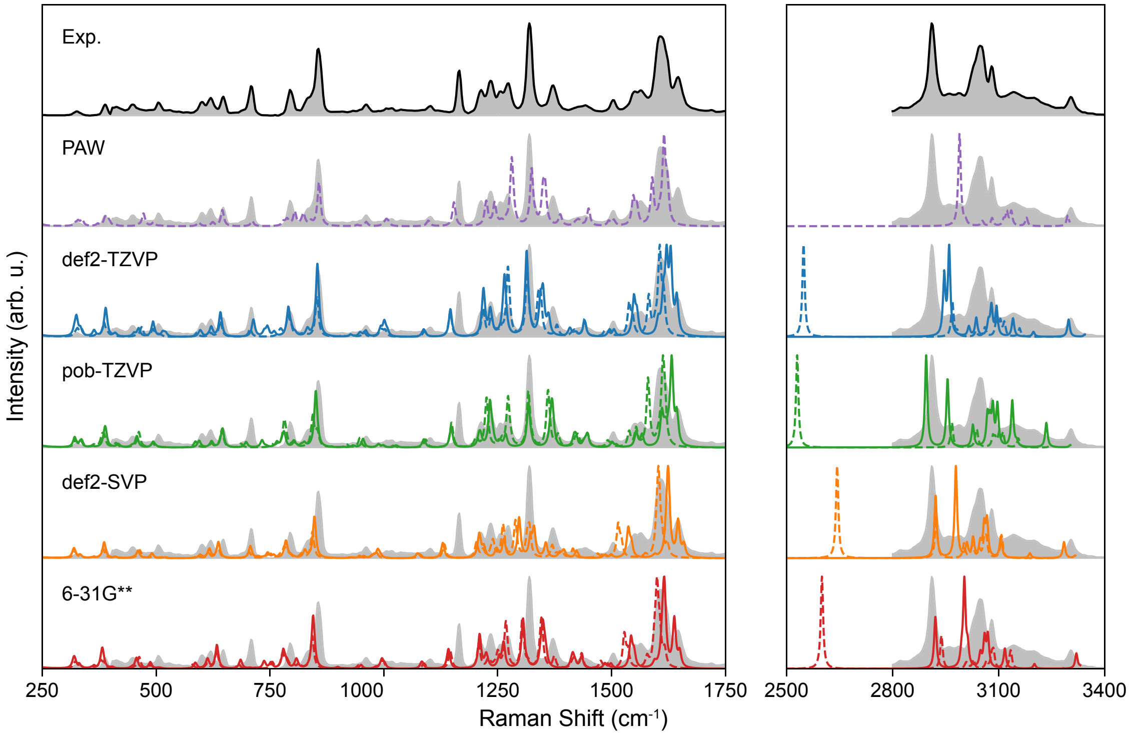

Figure S10: The Raman spectra of FI with anharmonic corrections. (left) from 250 to 1750 cm^{-1} and (right) from 2500 to 3400 cm^{-1}. Spectra in solid lines are obtained by PBE0, while spectra in dashed lines are obtained by PBE. Intensities are normalized to the highest peak of each plot. Experimental spectra adapted with permission from ref. 18. Copyright 2012 American Chemical Society.

For medium-frequency Raman spectra between 250 and 1750 cm^{-1}, differences in spectra based on various basis sets are negligible, suggesting that frequencies of the bending and heavy element stretching modes are properly reproduced after scaling. Hamiltonians lead

to similar results after frequency scaling, with limited discrepancies beyond 1500 cm^{-1}, indicating that a finer tuning of the scaling factors might be needed to better overlap with the experiment, which is, however, beyond the scope of this work.

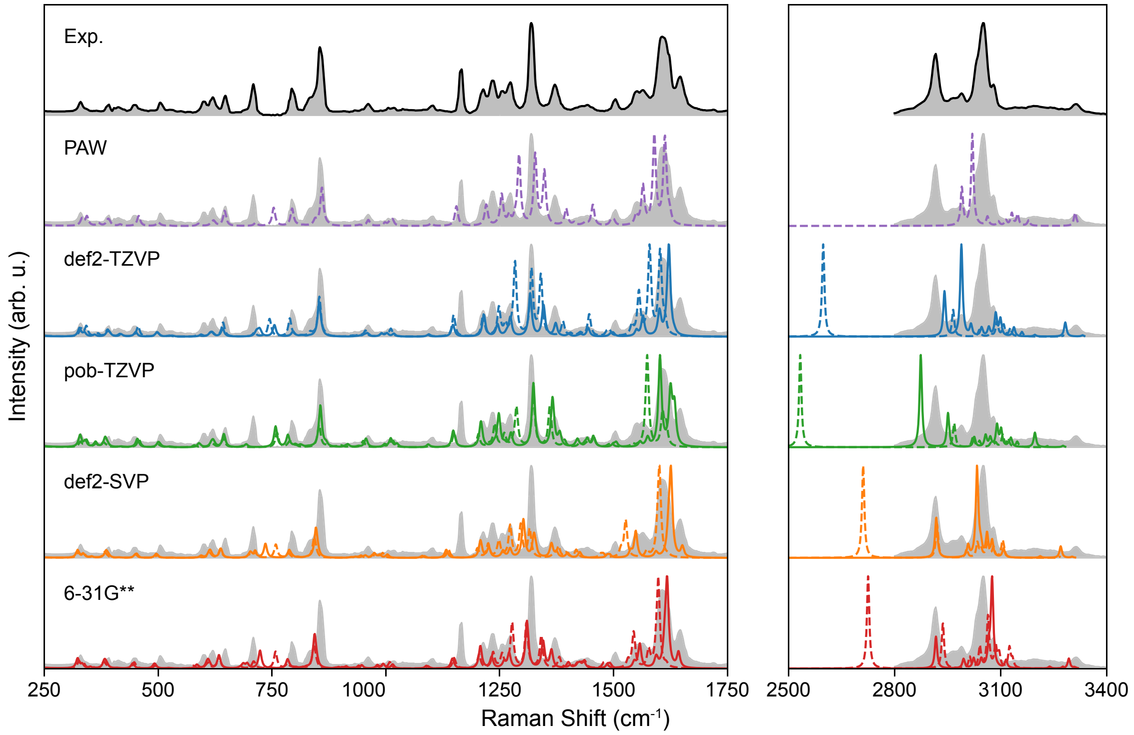

Figure S11: The Raman spectra of FII with anharmonic corrections. (left) from 250 to 1750 cm^{-1} and (right) from 2500 to 3400 cm^{-1}. Spectra in solid lines are obtained by PBE0, while those in dashed lines are obtained by PBE. Intensities are normalized to the highest peak of each plot. Experimental spectra adapted with permission from ref. 18. Copyright 2012 American Chemical Society.

In contrast, non-negligible deviations from the reference manifest in X−H stretching modes, even with corrections of anharmonicity. ν^{aha}(OH) and ν^{aha}(NH) are systematically underestimated by PBE, where, ironically, ν^{ha}(OH) and ν^{ha}(NH) agree better with experiments due to error cancellations originating from the stiffening effect of harmonic approximation. For ν^{aha}(NH), PBE0 generally achieves good agreement with the experiment, as indicated by the peak around 3300 cm^{-1}. The peak with the highest intensity, which corresponds to ν^{aha}(OH), exhibits stronger correlations with basis set and system, where spectra of smaller basis sets and FII agree better with experiments.

To summarize, the difficulties encountered in correctly reproducing the high-frequency vibrational spectra illustrate the complicated nature of X−H stretching modes and hydrogen-bonded systems, where multiple sources of error significantly influence the predicted frequencies and using a uniform scale factor is found to be inadequate. Adopting more advanced level of theory, such as double-hybrid functionals or high-order terms of the PES might be useful for a better description of the high-frequency modes.

S2.7 CPU time

All calculations are performed on the ARCHER2 UK National Supercomputing Service (https://www.archer2.ac.uk) with 128 cores (2×AMD EPYC 7742 64-core 2.25GHz processors) per node.[25]

Table S5: Time consumption

**Polymorph**	**Hamiltonian**	**Basis Set**	t_{CPU}(s)	n_{CYC}	n_{core}
form I	PBE0	6-31G**	171	14	256
		def2-SVP	447	14	512
		pob-TZVP	165	14	1024
		def2-TZVP	1653	13	1024
	PBE	6-31G**	105	13	128
		def2-SVP	248	15	256
		pob-TZVP	86	13	1024
		def2-TZVP	748	15	1024
		PAW	275	24	128
form II	PBE0	6-31G**	132	14	512
		def2-SVP	282	13	1024
		pob-TZVP	202	15	1024
		def2-TZVP	1144	16	1536
	PBE	6-31G**	100	15	256
		def2-SVP	153	15	768
		pob-TZVP	141	14	1024
		def2-TZVP	1361	18	1024
		PAW	544	26	512

Practically, to reduce the cost of phonon calculations, the converged density matrix based on equilibrium geometry is adopted as the initial guess of displaced geometries. The computational parameters and CPU time reported in **Table S5** are based on these preliminary SCF

steps, including the CPU time t_{CPU}, the number of SCF iterations n_{SCF}, and the number of cores n_{core}. The finite-displacement scheme of this study (**Methodology**, manuscript) requires 120 total energy and gradient calculations for both FI and FII. However, the overall computational cost is expected to be higher than $120 \times t_{\text{CPU}}$, since all displacements reduce the space group symmetry to $P1$. This increases the number of irreducible atoms to 80 for FI and 160 for FII, instead of 20 for the non-displaced geometries.

S2.8 Vibrational frequencies

Non-translational harmonic vibrational frequencies sorted with reference to PAW/PBE are listed in **Table S6** and **Table S7** respectively for FI and FII. Frequencies are calculated based on optimized geometries as reported in **Table S3** and sorted by **Equation 2** of the manuscript.

Table S6: Harmonic vibrational frequencies of form I paracetamol (Unit: cm^{-1})

#	PAW	6-31G**		def2-SVP		pob-TZVP		def2-TZVP	
	PBE	PBE0	PBE	PBE0	PBE	PBE0	PBE	PBE0	PBE
4	31	38	42	34	39	50	53	36	37
5	40	39	44	35	37	44	41	44	40
6	48	44	48	48	51	41	41	56	51
7	55	43	39	40	41	51	51	55	51
8	62	59	61	59	56	74	70	66	61
9	62	61	65	55	61	44	47	63	58
10	64	59	61	59	59	62	70	63	64
11	66	64	70	60	65	68	69	63	61
12	74	70	72	66	68	78	84	73	74
13	74	74	73	73	70	89	89	78	76
14	79	73	73	77	74	86	89	81	79
15	80	79	81	80	80	98	98	87	84
16	88	85	84	86	84	104	109	95	89
17	95	93	94	86	90	107	109	95	96
18	95	87	90	87	85	93	96	91	95
19	102	99	101	97	95	98	100	101	103
20	103	104	106	101	100	136	144	108	103
21	104	99	105	101	101	127	130	103	103
22	105	100	105	104	103	99	102	104	109
23	106	101	101	100	97	130	131	104	106
24	113	108	115	98	115	170	179	118	111
25	114	113	130	95	127	106	108	115	123

#	PAW	6-31G**		def2-SVP		pob-TZVP		def2-TZVP	
	PBE	PBE0	PBE	PBE0	PBE	PBE0	PBE	PBE0	PBE
26	115	107	116	92	111	143	148	115	118
27	116	111	111	112	108	137	144	120	116
28	117	111	127	103	123	126	137	122	122
29	131	131	138	128	131	110	112	133	134
30	142	141	146	145	144	179	183	144	143
31	143	150	154	135	147	179	185	155	143
32	152	161	162	135	159	185	187	168	148
33	162	164	169	138	166	131	133	175	161
34	162	164	174	135	165	111	115	170	164
35	168	147	151	149	146	150	157	157	165
36	175	159	162	159	158	161	166	167	173
37	180	179	185	174	180	165	169	183	180
38	183	184	189	175	181	153	154	187	187
39	207	207	205	207	202	210	210	211	207
40	211	209	207	211	205	228	219	214	211
41	217	221	215	220	212	224	217	224	217
42	219	220	215	223	215	232	225	224	219
43	235	224	224	220	214	228	227	228	232
44	237	225	225	221	215	216	223	229	233
45	322	333	323	331	321	331	321	333	323
46	323	335	326	333	323	334	325	334	324
47	329	338	329	335	325	335	327	339	330
48	329	339	331	337	327	336	327	339	331
49	337	348	339	348	338	348	339	348	339
50	340	350	341	349	339	349	340	349	341
51	372	377	369	377	366	375	366	379	372
52	373	380	371	378	366	375	365	381	373
53	388	401	384	404	386	403	384	405	388
54	389	404	387	407	389	400	381	406	389
55	392	403	389	405	389	401	386	405	392
56	396	403	391	404	388	406	389	407	396
57	410	423	409	428	412	434	414	427	409
58	411	425	410	430	413	436	417	428	410
59	412	424	408	428	411	438	417	431	411
60	413	425	409	430	411	439	418	432	412
61	461	469	459	472	459	466	454	471	460
62	463	472	461	474	460	467	455	472	463
63	472	481	470	482	469	477	466	481	472
64	472	480	470	482	469	477	466	481	472
65	494	512	496	514	498	514	496	514	497
66	494	512	497	514	498	517	501	514	496
67	495	511	495	513	497	515	497	514	497
68	496	512	496	514	498	520	504	515	497
69	509	535	513	545	520	539	513	537	510
70	509	531	511	542	517	539	512	536	509
71	511	537	515	546	520	543	517	540	512

#	PAW	6-31G**		def2-SVP		pob-TZVP		def2-TZVP	
	PBE	PBE0	PBE	PBE0	PBE	PBE0	PBE	PBE0	PBE
72	514	536	516	547	521	547	520	543	515
73	593	616	589	624	596	620	591	621	593
74	593	615	587	624	596	617	588	621	593
75	595	619	591	625	597	623	593	623	595
76	595	618	590	626	598	618	589	623	595
77	618	639	616	640	616	644	622	642	619
78	619	640	618	642	618	644	622	642	620
79	622	643	620	643	620	647	625	645	623
80	624	645	622	646	622	648	626	647	625
81	644	666	642	666	641	670	647	668	645
82	645	665	642	666	641	671	648	668	645
83	645	666	642	666	641	671	647	669	646
84	646	666	643	666	642	673	650	669	646
85	710	719	691	737	710	726	692	741	711
86	711	723	693	741	712	727	693	744	711
87	711	721	692	739	711	727	692	742	711
88	711	721	691	740	711	727	692	744	712
89	779	829	766	839	751	797	760	769	775
90	783	824	767	832	759	810	764	832	778
91	784	826	759	835	752	826	759	769	774
92	784	825	769	837	760	766	762	775	778
93	787	819	767	821	789	814	772	824	780
94	792	820	786	822	788	826	777	824	791
95	792	821	784	824	789	810	778	836	791
96	792	820	786	823	788	817	780	827	791
97	804	742	790	758	759	841	787	839	796
98	806	743	789	760	796	828	809	827	804
99	812	754	789	770	771	835	813	787	811
100	819	848	798	864	796	854	811	776	817
101	819	775	799	781	767	761	792	778	802
102	823	791	805	793	779	773	791	792	802
103	824	782	800	787	798	810	793	837	802
104	824	775	803	780	803	764	808	776	813
105	830	755	814	770	825	837	845	867	826
106	835	853	819	866	825	860	855	870	831
107	838	855	822	869	830	865	849	873	836
108	840	850	826	865	828	860	860	869	836
109	857	888	856	887	852	887	854	890	859
110	858	888	856	887	851	887	851	891	860
111	861	891	859	889	854	891	863	893	862
112	861	891	859	889	854	891	863	892	862
113	916	943	899	968	921	942	886	962	912
114	918	947	902	970	922	945	888	965	914
115	919	942	901	967	920	948	892	966	915
116	920	946	902	970	922	950	893	967	916
117	941	959	914	986	938	971	925	987	936

#	PAW	6-31G**		def2-SVP		pob-TZVP		def2-TZVP	
	PBE	PBE0	PBE	PBE0	PBE	PBE0	PBE	PBE0	PBE
118	943	964	917	990	940	974	926	990	938
119	947	966	915	991	941	984	934	996	943
120	950	971	920	994	944	990	938	1003	946
121	959	997	956	999	954	993	953	998	961
122	960	997	957	998	954	994	954	1000	962
123	961	999	959	1000	956	995	955	1001	964
124	963	1000	960	1001	957	996	956	1003	965
125	998	1033	996	1023	984	1027	992	1034	998
126	999	1032	996	1022	982	1032	997	1034	999
127	999	1033	996	1022	983	1030	994	1035	999
128	999	1034	996	1024	984	1032	998	1035	999
129	1004	1045	1008	1030	990	1040	1003	1042	1004
130	1005	1046	1008	1031	990	1042	1005	1043	1004
131	1005	1047	1009	1033	991	1041	1006	1044	1004
132	1006	1048	1011	1033	992	1041	1003	1044	1007
133	1009	1055	1014	1040	994	1072	1031	1051	1009
134	1009	1054	1014	1039	993	1071	1028	1051	1011
135	1010	1053	1014	1038	994	1069	1028	1050	1011
136	1010	1057	1015	1042	996	1072	1030	1053	1010
137	1097	1138	1104	1123	1088	1133	1099	1133	1097
138	1099	1141	1107	1127	1091	1135	1101	1137	1100
139	1101	1144	1108	1129	1093	1139	1105	1140	1103
140	1103	1146	1110	1132	1095	1141	1107	1142	1105
141	1154	1200	1163	1182	1143	1196	1157	1195	1154
142	1155	1202	1165	1182	1145	1197	1159	1196	1156
143	1157	1205	1167	1185	1147	1198	1160	1197	1158
144	1158	1207	1168	1189	1149	1200	1161	1200	1160
145	1224	1273	1227	1265	1215	1260	1210	1270	1227
146	1226	1273	1226	1267	1215	1264	1214	1272	1228
147	1226	1274	1227	1267	1216	1263	1213	1273	1228
148	1226	1275	1230	1269	1218	1262	1212	1272	1229
149	1243	1288	1248	1276	1229	1285	1235	1286	1245
150	1247	1290	1251	1277	1230	1286	1234	1288	1249
151	1250	1313	1262	1304	1247	1291	1237	1290	1253
152	1252	1294	1254	1281	1234	1297	1248	1293	1254
153	1258	1291	1252	1276	1230	1302	1261	1308	1258
154	1261	1312	1262	1301	1247	1307	1265	1308	1261
155	1268	1318	1269	1306	1252	1311	1270	1315	1268
156	1269	1318	1270	1306	1252	1309	1269	1315	1269
157	1281	1328	1287	1322	1274	1321	1283	1319	1282
158	1283	1333	1290	1327	1277	1322	1281	1324	1285
159	1285	1329	1289	1323	1276	1322	1288	1321	1286
160	1286	1333	1293	1329	1280	1323	1288	1324	1288
161	1325	1373	1324	1357	1301	1373	1328	1370	1325
162	1330	1381	1332	1365	1309	1378	1332	1375	1331
163	1331	1380	1332	1365	1309	1383	1336	1377	1332

#	PAW	6-31G**		def2-SVP		pob-TZVP		def2-TZVP	
	PBE	PBE0	PBE	PBE0	PBE	PBE0	PBE	PBE0	PBE
164	1333	1382	1337	1363	1312	1380	1335	1376	1334
165	1349	1415	1365	1384	1327	1427	1370	1401	1349
166	1351	1415	1369	1384	1331	1420	1371	1401	1352
167	1352	1416	1367	1388	1330	1429	1370	1403	1352
168	1354	1419	1368	1392	1333	1429	1373	1407	1354
169	1387	1424	1395	1418	1383	1422	1393	1419	1390
170	1388	1425	1396	1419	1384	1420	1394	1421	1392
171	1389	1426	1397	1420	1383	1425	1396	1422	1392
172	1390	1430	1398	1422	1383	1434	1397	1425	1394
173	1417	1482	1434	1447	1392	1480	1433	1465	1419
174	1418	1482	1434	1448	1392	1472	1427	1465	1420
175	1419	1484	1434	1450	1393	1482	1437	1466	1422
176	1422	1488	1439	1451	1395	1478	1432	1469	1424
177	1426	1487	1436	1460	1403	1499	1453	1477	1428
178	1426	1486	1435	1459	1403	1500	1448	1478	1429
179	1428	1489	1440	1459	1402	1496	1452	1479	1429
180	1430	1493	1443	1462	1405	1502	1454	1483	1432
181	1442	1503	1450	1484	1423	1497	1448	1496	1444
182	1445	1504	1451	1488	1427	1509	1458	1498	1447
183	1449	1510	1457	1490	1429	1508	1460	1504	1451
184	1450	1508	1457	1489	1429	1508	1459	1502	1452
185	1493	1562	1499	1553	1484	1563	1501	1556	1495
186	1494	1562	1499	1553	1484	1565	1503	1557	1497
187	1505	1576	1513	1568	1497	1575	1511	1569	1507
188	1505	1576	1512	1569	1498	1575	1510	1570	1508
189	1548	1622	1552	1608	1529	1620	1551	1615	1551
190	1552	1626	1556	1612	1533	1623	1555	1619	1555
191	1554	1627	1558	1613	1534	1628	1561	1620	1557
192	1555	1629	1560	1615	1536	1633	1566	1622	1558
193	1589	1676	1601	1669	1589	1680	1595	1670	1594
194	1589	1677	1602	1669	1589	1679	1592	1671	1594
195	1599	1695	1619	1697	1615	1683	1602	1683	1604
196	1599	1726	1634	1727	1630	1683	1599	1675	1604
197	1606	1678	1606	1670	1590	1686	1614	1674	1609
198	1609	1679	1606	1671	1591	1691	1617	1687	1613
199	1614	1697	1622	1698	1617	1701	1624	1702	1617
200	1614	1722	1631	1723	1626	1694	1620	1699	1616
201	1614	1702	1624	1703	1618	1699	1623	1692	1617
202	1615	1700	1625	1701	1619	1701	1625	1690	1618
203	1620	1728	1638	1729	1634	1705	1628	1706	1625
204	1622	1735	1642	1736	1638	1713	1630	1713	1627
205	2985	3072	2984	3057	2952	3081	2991	3071	2986
206	2988	3072	2984	3058	2952	3081	2993	3072	2991
207	2989	3071	2984	3057	2951	3081	2991	3071	2992
208	2989	3072	2984	3058	2952	3081	2991	3072	2992
209	2990	3357	3039	3327	3042	3275	2975	3325	2989

#	PAW	6-31G**		def2-SVP		pob-TZVP		def2-TZVP	
	PBE	PBE0	PBE	PBE0	PBE	PBE0	PBE	PBE0	PBE
210	2995	3358	3040	3329	3043	3274	2975	3327	2996
211	2999	3363	3049	3333	3050	3279	2983	3331	2998
212	3007	3367	3059	3337	3058	3285	2989	3335	3006
213	3054	3149	3054	3150	3031	3155	3059	3144	3059
214	3054	3149	3055	3150	3031	3155	3059	3144	3059
215	3054	3150	3054	3150	3031	3155	3059	3144	3059
216	3055	3150	3051	3150	3031	3155	3059	3145	3060
217	3082	3168	3076	3169	3054	3198	3108	3166	3085
218	3082	3168	3076	3169	3055	3198	3108	3166	3085
219	3082	3168	3076	3169	3052	3198	3108	3166	3085
220	3082	3168	3076	3169	3054	3198	3108	3166	3085
221	3115	3206	3113	3190	3075	3206	3114	3202	3118
222	3115	3206	3113	3190	3075	3206	3115	3202	3118
223	3115	3206	3113	3190	3075	3206	3115	3202	3118
224	3115	3206	3113	3190	3075	3206	3115	3202	3118
225	3123	3218	3128	3202	3089	3214	3126	3211	3129
226	3123	3218	3128	3202	3089	3214	3126	3211	3129
227	3123	3218	3128	3202	3089	3214	3126	3211	3129
228	3123	3218	3128	3202	3089	3214	3126	3211	3129
229	3136	3228	3133	3210	3095	3228	3137	3226	3140
230	3136	3229	3133	3210	3095	3228	3137	3226	3140
231	3136	3229	3133	3210	3095	3228	3137	3226	3141
232	3136	3229	3133	3210	3095	3228	3137	3226	3140
233	3178	3274	3179	3247	3137	3268	3179	3269	3182
234	3178	3274	3179	3247	3137	3268	3179	3269	3182
235	3179	3279	3181	3251	3138	3271	3180	3275	3184
236	3179	3279	3181	3251	3139	3272	3181	3275	3184
237	3295	3498	3330	3465	3296	3436	3260	3481	3302
238	3295	3498	3330	3466	3297	3437	3260	3482	3303
239	3299	3501	3333	3470	3299	3440	3264	3485	3306
240	3302	3502	3335	3471	3301	3442	3267	3486	3308

Table S7: Harmonic vibrational frequencies of form II paracetamol (Unit: cm^{-1})

#	PAW	6-31G**		def2-SVP		pob-TZVP		def2-TZVP	
	**PBE**	**PBE0**	**PBE**	**PBE0**	**PBE**	**PBE0**	**PBE**	**PBE0**	**PBE**
4	20	31	29	27	26	19	13	29	29
5	21	27	28	25	26	24	26	25	26
6	28	28	28	27	27	22	23	24	26
7	30	28	28	25	24	18	18	27	27
8	38	36	36	35	34	31	31	35	36
9	46	49	48	46	46	44	45	50	50
10	47	49	47	45	43	50	47	50	50
11	48	49	49	48	47	49	49	51	52
12	53	57	56	55	53	59	56	54	54
13	54	52	53	51	50	55	55	54	55
14	57	56	57	56	55	60	59	56	57
15	59	59	61	57	56	58	56	55	58
16	63	64	66	66	66	61	61	67	67
17	63	63	64	61	60	69	67	65	65
18	64	60	63	61	61	63	65	61	63
19	66	68	69	70	69	63	63	72	71
20	71	72	76	74	74	70	69	73	75
21	72	72	73	68	68	74	73	72	74
22	74	76	75	75	73	75	77	76	79
23	76	74	71	76	73	77	77	79	78
24	77	78	80	80	79	73	73	80	80
25	81	77	75	74	72	82	81	81	84
26	81	78	80	80	79	87	87	80	82
27	82	75	76	74	72	81	81	81	83
28	85	85	86	86	84	87	86	87	87
29	87	89	87	88	84	87	87	91	92
30	88	88	91	88	86	90	91	89	94
31	89	89	87	89	86	88	88	93	92
32	90	95	96	90	89	94	96	94	97
33	93	96	99	94	93	101	98	92	97
34	94	92	95	93	92	101	103	94	98
35	99	100	104	99	100	99	100	100	103
36	103	98	104	96	95	104	105	104	106
37	103	102	102	101	98	103	103	103	106
38	103	104	105	98	96	102	104	107	106
39	104	105	109	103	103	94	103	105	110
40	105	99	103	101	101	109	110	103	106
41	106	103	97	102	100	111	114	109	110
42	107	112	112	111	109	114	116	109	112
43	108	100	102	102	100	110	112	100	110
44	113	118	121	115	116	121	122	115	118
45	115	113	115	112	109	116	117	119	119
46	116	115	118	114	112	120	121	116	119

#	PAW	6-31G**		def2-SVP		pob-TZVP		def2-TZVP	
	PBE	PBE0	PBE	PBE0	PBE	PBE0	PBE	PBE0	PBE
47	117	117	119	116	117	123	124	118	121
48	117	122	123	118	118	123	123	120	122
49	119	119	118	119	116	121	124	121	124
50	121	122	121	123	120	126	126	125	123
51	123	132	134	130	129	128	131	117	131
52	127	123	125	122	120	130	132	129	131
53	130	124	128	121	119	132	135	128	133
54	131	133	133	131	129	141	140	132	136
55	132	129	133	126	125	134	137	130	137
56	132	131	131	128	125	139	140	136	138
57	133	135	139	134	134	132	135	133	142
58	134	139	142	138	135	142	145	133	143
59	136	138	142	138	136	134	137	134	144
60	141	130	133	129	127	144	146	137	143
61	142	142	143	139	135	149	148	140	150
62	143	160	165	156	159	150	151	142	152
63	143	141	143	138	135	153	155	147	149
64	144	143	145	140	139	150	152	145	151
65	154	140	141	138	134	159	160	152	160
66	156	164	167	163	163	163	160	158	165
67	158	171	174	167	168	168	167	163	171
68	158	168	169	163	161	171	171	161	167
69	165	177	181	170	172	179	180	166	174
70	165	176	185	173	179	176	176	165	174
71	169	187	194	176	183	178	179	171	177
72	172	192	196	180	182	188	189	177	183
73	181	198	204	186	190	189	188	187	187
74	182	196	199	185	186	190	188	187	187
75	189	198	196	190	188	197	196	193	192
76	190	201	203	193	193	201	201	195	196
77	199	204	199	204	197	202	196	205	200
78	206	207	208	201	200	211	210	207	208
79	206	209	211	201	201	209	210	207	209
80	210	209	208	205	199	216	212	214	213
81	213	220	221	219	215	218	213	220	215
82	217	221	220	220	211	224	221	224	221
83	219	223	218	222	213	227	221	226	221
84	219	226	223	222	216	227	220	226	221
85	220	222	218	221	212	227	220	225	222
86	222	216	214	213	207	228	227	222	225
87	226	224	219	223	215	236	233	229	228
88	230	232	226	231	222	241	234	237	232
89	326	335	325	332	321	338	326	335	326
90	326	335	325	333	322	336	327	335	326
91	326	336	326	334	323	336	326	336	326
92	327	336	327	334	323	336	324	336	327

#	PAW	6-31G**		def2-SVP		pob-TZVP		def2-TZVP	
	PBE	PBE0	PBE	PBE0	PBE	PBE0	PBE	PBE0	PBE
93	333	340	331	338	328	343	332	341	333
94	333	340	331	338	327	342	333	341	333
95	333	342	333	340	330	342	331	343	334
96	334	343	334	341	330	341	333	344	334
97	343	350	341	351	340	354	344	352	344
98	343	349	340	349	339	353	346	351	345
99	344	350	341	351	340	354	345	353	345
100	345	349	341	350	339	356	348	352	346
101	368	368	360	369	357	378	368	373	368
102	369	371	363	371	360	376	366	375	370
103	370	369	362	370	359	378	366	376	371
104	372	372	364	372	361	381	371	377	373
105	385	401	385	402	385	394	377	404	387
106	388	401	385	402	385	400	387	404	390
107	390	403	387	405	386	400	383	408	392
108	391	404	387	405	386	401	382	409	392
109	393	408	392	410	392	405	387	413	396
110	394	408	392	409	391	405	390	411	396
111	394	406	390	407	389	406	391	411	396
112	395	405	390	407	390	406	392	411	397
113	416	430	413	433	416	429	411	435	417
114	416	430	413	433	416	428	411	435	417
115	417	432	416	434	417	435	417	437	420
116	417	432	416	434	417	435	418	436	420
117	424	438	422	440	424	441	424	443	427
118	424	439	422	440	424	442	424	444	427
119	426	440	424	443	426	445	428	445	428
120	426	440	424	443	426	445	428	445	429
121	443	458	445	462	448	460	447	459	447
122	444	458	445	462	448	461	447	459	447
123	449	462	449	466	452	467	454	464	452
124	451	464	451	467	454	468	455	466	454
125	457	467	454	471	457	473	461	470	460
126	457	467	454	471	457	473	461	470	460
127	459	469	456	473	459	475	463	472	461
128	459	470	457	474	460	476	463	473	463
129	499	516	500	518	501	521	504	517	501
130	500	517	501	519	503	522	505	518	501
131	501	519	502	521	503	522	503	520	502
132	501	519	502	520	503	523	506	520	503
133	504	519	503	521	505	526	509	521	505
134	504	520	504	522	505	527	510	521	506
135	504	519	504	522	505	525	507	522	506
136	509	523	507	526	508	530	512	526	510
137	509	538	515	543	521	532	510	540	515
138	510	539	516	545	523	534	512	541	516

#	PAW	6-31G**		def2-SVP		pob-TZVP		def2-TZVP	
	PBE	PBE0	PBE	PBE0	PBE	PBE0	PBE	PBE0	PBE
139	521	547	524	553	529	548	525	550	526
140	521	549	526	554	532	544	520	551	526
141	521	547	524	552	529	550	526	550	526
142	522	550	527	555	533	547	523	552	527
143	524	551	528	556	533	553	530	555	530
144	525	551	528	556	533	553	529	555	531
145	588	615	586	622	593	614	583	619	592
146	588	615	586	622	593	615	585	620	592
147	589	616	587	623	594	616	586	621	593
148	590	618	588	624	595	617	587	622	594
149	593	618	589	625	596	618	589	624	597
150	594	620	591	626	597	620	590	625	598
151	594	619	591	626	597	618	590	625	599
152	595	621	591	626	598	620	591	626	599
153	616	638	614	640	616	642	618	640	617
154	617	638	616	640	616	643	620	640	618
155	617	639	616	641	617	644	619	641	619
156	618	639	617	641	618	644	620	642	620
157	621	641	619	642	619	646	624	643	623
158	621	641	619	643	620	647	624	644	623
159	623	642	620	643	621	649	626	645	625
160	625	644	621	645	622	650	627	647	626
161	645	666	643	667	642	672	648	669	646
162	645	667	643	667	642	671	648	669	646
163	645	666	642	667	643	671	648	669	646
164	645	665	642	667	642	672	648	669	647
165	646	666	643	667	643	672	648	669	647
166	647	668	644	670	644	672	649	670	648
167	647	667	644	669	644	673	650	670	648
168	647	667	645	669	644	673	650	670	648
169	709	719	688	732	703	716	684	744	713
170	710	720	689	732	704	717	684	745	713
171	713	721	690	734	706	721	688	747	716
172	714	721	690	735	706	721	689	748	717
173	714	719	690	734	706	723	690	747	717
174	715	723	691	735	707	723	691	751	717
175	715	722	691	736	707	725	692	749	718
176	716	722	691	736	708	725	692	749	719
177	747	714	716	734	714	781	760	744	744
178	750	754	726	743	720	784	761	755	748
179	753	730	721	743	719	787	767	754	750
180	753	761	720	740	719	790	767	753	750
181	754	732	723	747	721	790	766	755	752
182	755	741	728	753	723	792	767	760	753
183	755	739	728	750	723	791	769	759	753
184	758	756	728	766	725	796	772	761	756

#	PAW	6-31G**		def2-SVP		pob-TZVP		def2-TZVP	
	PBE	PBE0	PBE	PBE0	PBE	PBE0	PBE	PBE0	PBE
185	790	821	792	845	808	821	788	848	792
186	793	842	764	845	808	826	790	849	795
187	793	838	780	842	777	818	801	804	795
188	794	825	796	824	794	818	794	830	795
189	795	838	776	842	773	822	790	847	797
190	796	822	794	824	794	824	797	828	798
191	798	829	794	831	798	833	791	840	801
192	798	826	796	825	795	823	802	830	800
193	800	823	794	825	795	827	800	829	802
194	801	829	794	831	797	833	805	839	803
195	803	842	806	823	793	834	805	829	807
196	805	845	820	848	812	833	796	854	808
197	805	825	796	827	795	833	805	832	808
198	806	844	804	848	811	836	800	853	810
199	817	868	830	857	820	847	809	865	821
200	818	859	820	865	827	851	811	869	820
201	818	869	832	872	834	847	810	875	822
202	818	859	805	865	827	851	811	869	820
203	824	854	812	859	821	861	821	866	827
204	825	854	812	872	833	861	821	875	828
205	830	865	824	872	833	856	826	878	829
206	831	865	806	872	833	866	825	877	830
207	833	871	829	878	838	861	821	883	838
208	833	871	831	878	839	861	821	883	838
209	842	728	768	747	764	861	876	783	838
210	843	762	824	770	765	858	859	789	841
211	844	728	763	765	761	866	875	783	841
212	845	720	769	770	766	864	876	788	841
213	849	775	783	787	780	871	859	808	847
214	849	787	792	796	788	873	857	816	847
215	856	888	856	886	852	881	860	890	853
216	860	887	855	886	851	892	860	890	861
217	860	890	858	888	853	894	862	892	861
218	861	888	856	886	852	893	859	891	862
219	861	770	811	780	811	892	888	798	860
220	862	891	858	889	854	896	879	894	864
221	865	894	861	891	856	899	864	896	866
222	867	784	816	790	814	889	895	848	863
223	872	893	861	891	856	906	895	896	871
224	874	892	860	890	855	905	897	895	872
225	923	950	903	966	924	949	902	972	924
226	923	950	903	967	924	949	903	973	925
227	927	954	907	969	926	952	906	976	928
228	928	955	907	970	927	954	908	977	929
229	933	960	910	978	934	956	911	983	933
230	933	961	910	978	934	957	912	983	934

#	PAW	6-31G**		def2-SVP		pob-TZVP		def2-TZVP	
	PBE	PBE0	PBE	PBE0	PBE	PBE0	PBE	PBE0	PBE
231	934	960	910	977	934	955	909	982	934
232	934	960	911	976	934	955	909	982	934
233	941	975	925	991	945	978	928	993	945
234	942	975	925	992	945	979	928	994	945
235	942	977	930	992	947	974	923	994	948
236	943	977	930	993	947	974	923	995	948
237	948	981	931	995	950	978	928	997	951
238	949	982	931	1001	951	978	929	1002	952
239	951	985	935	1001	952	982	933	1004	954
240	951	986	936	1001	955	982	933	1004	955
241	959	994	953	997	953	995	956	998	961
242	960	995	954	996	954	996	954	998	962
243	960	995	954	1001	955	996	957	1003	962
244	961	995	954	997	956	996	957	998	962
245	962	996	955	999	955	997	958	1000	963
246	962	996	955	999	955	997	958	1001	963
247	962	996	955	999	955	998	958	1001	963
248	962	997	956	1000	955	999	959	1001	964
249	997	1031	994	1019	979	1036	1001	1031	997
250	997	1030	994	1020	981	1036	1000	1032	997
251	998	1031	995	1021	981	1038	1002	1033	998
252	998	1032	996	1020	980	1039	1004	1033	998
253	1000	1033	996	1022	982	1038	1002	1036	1001
254	1000	1034	998	1022	982	1051	1014	1036	1001
255	1001	1035	999	1023	983	1050	1014	1036	1001
256	1002	1036	999	1023	983	1051	1014	1037	1003
257	1004	1043	1006	1031	992	1041	1005	1041	1005
258	1005	1042	1006	1029	991	1050	1014	1041	1006
259	1005	1042	1007	1030	992	1043	1007	1042	1006
260	1005	1043	1006	1030	992	1042	1005	1042	1006
261	1006	1045	1008	1032	992	1054	1017	1044	1008
262	1007	1057	1015	1040	994	1059	1017	1052	1010
263	1007	1057	1015	1040	994	1059	1017	1052	1010
264	1007	1058	1016	1040	993	1059	1017	1053	1011
265	1008	1044	1009	1032	992	1054	1016	1045	1009
266	1008	1046	1009	1032	995	1055	1019	1044	1009
267	1008	1046	1009	1032	993	1054	1017	1045	1010
268	1009	1058	1016	1041	995	1060	1018	1053	1011
269	1015	1067	1024	1050	1002	1068	1024	1061	1019
270	1015	1068	1025	1051	1003	1068	1025	1062	1019
271	1015	1068	1025	1051	1003	1068	1024	1062	1019
272	1015	1068	1025	1051	1003	1068	1026	1061	1019
273	1099	1145	1109	1130	1095	1140	1105	1139	1103
274	1099	1145	1109	1130	1096	1139	1104	1138	1103
275	1100	1145	1110	1130	1096	1139	1104	1138	1103
276	1100	1146	1110	1131	1096	1140	1105	1139	1104

#	PAW	6-31G**		def2-SVP		pob-TZVP		def2-TZVP	
	**PBE**	**PBE0**	**PBE**	**PBE0**	**PBE**	**PBE0**	**PBE**	**PBE0**	**PBE**
277	1100	1146	1110	1130	1095	1143	1108	1139	1103
278	1101	1147	1111	1131	1097	1143	1109	1140	1104
279	1103	1150	1114	1134	1100	1146	1112	1143	1107
280	1104	1150	1114	1135	1102	1147	1112	1143	1108
281	1155	1205	1167	1185	1148	1196	1158	1196	1157
282	1155	1205	1167	1185	1148	1196	1159	1196	1158
283	1155	1205	1168	1185	1148	1197	1159	1197	1159
284	1156	1205	1168	1185	1148	1197	1160	1197	1158
285	1156	1206	1168	1186	1149	1197	1160	1197	1158
286	1156	1206	1169	1186	1149	1198	1161	1198	1160
287	1158	1208	1171	1188	1151	1200	1162	1200	1161
288	1158	1208	1171	1188	1151	1200	1162	1200	1161
289	1220	1271	1224	1264	1213	1258	1209	1267	1223
290	1220	1270	1223	1264	1212	1260	1212	1267	1223
291	1221	1270	1224	1265	1213	1261	1213	1268	1223
292	1222	1272	1225	1265	1215	1261	1214	1268	1225
293	1223	1272	1225	1266	1215	1262	1215	1270	1226
294	1225	1273	1226	1268	1218	1265	1219	1271	1227
295	1226	1274	1227	1269	1217	1266	1220	1273	1229
296	1227	1274	1227	1269	1217	1265	1218	1273	1229
297	1242	1291	1249	1280	1233	1286	1233	1289	1245
298	1243	1295	1251	1307	1236	1288	1233	1293	1246
299	1245	1294	1252	1323	1236	1290	1236	1293	1249
300	1249	1297	1254	1314	1237	1295	1241	1298	1253
301	1252	1298	1257	1311	1239	1297	1242	1299	1256
302	1252	1296	1257	1307	1253	1296	1243	1296	1256
303	1254	1297	1256	1284	1238	1299	1246	1298	1258
304	1254	1296	1255	1283	1237	1301	1249	1298	1258
305	1266	1316	1267	1283	1251	1315	1272	1311	1268
306	1268	1321	1270	1284	1257	1315	1271	1314	1269
307	1268	1321	1271	1311	1256	1314	1270	1316	1269
308	1268	1318	1269	1284	1240	1314	1273	1314	1270
309	1269	1318	1270	1306	1252	1311	1274	1313	1271
310	1269	1320	1271	1286	1256	1314	1274	1315	1271
311	1271	1322	1272	1283	1257	1310	1274	1314	1273
312	1271	1325	1275	1316	1261	1314	1270	1320	1273
313	1284	1329	1288	1312	1276	1329	1288	1323	1285
314	1285	1331	1290	1326	1278	1327	1288	1324	1287
315	1287	1333	1292	1328	1280	1327	1291	1325	1288
316	1288	1338	1295	1333	1286	1326	1289	1329	1290
317	1292	1333	1295	1328	1282	1328	1298	1325	1293
318	1292	1335	1296	1330	1284	1327	1296	1326	1293
319	1293	1336	1297	1332	1285	1329	1297	1328	1294
320	1295	1339	1300	1335	1288	1329	1298	1331	1297
321	1325	1373	1325	1356	1304	1377	1331	1369	1327
322	1328	1377	1329	1363	1309	1380	1334	1373	1330

#	PAW	6-31G**		def2-SVP		pob-TZVP		def2-TZVP	
	PBE	PBE0	PBE	PBE0	PBE	PBE0	PBE	PBE0	PBE
323	1329	1378	1329	1361	1308	1381	1335	1373	1330
324	1330	1380	1331	1363	1310	1383	1337	1375	1332
325	1331	1380	1332	1363	1311	1384	1338	1376	1333
326	1334	1384	1336	1368	1315	1389	1343	1379	1336
327	1336	1385	1339	1365	1314	1388	1345	1379	1337
328	1338	1388	1340	1369	1318	1393	1348	1382	1340
329	1348	1415	1360	1389	1327	1425	1369	1405	1350
330	1348	1415	1362	1387	1328	1424	1370	1403	1351
331	1349	1414	1362	1388	1329	1425	1371	1404	1351
332	1349	1416	1362	1388	1328	1426	1372	1405	1351
333	1349	1416	1363	1391	1330	1427	1372	1405	1352
334	1350	1416	1362	1391	1329	1424	1369	1405	1352
335	1350	1416	1361	1393	1329	1426	1371	1406	1352
336	1352	1419	1363	1396	1333	1427	1371	1410	1355
337	1393	1431	1400	1425	1395	1433	1398	1428	1397
338	1393	1431	1401	1425	1395	1435	1398	1428	1397
339	1394	1433	1400	1426	1383	1437	1400	1429	1398
340	1395	1433	1401	1428	1392	1437	1400	1430	1398
341	1395	1432	1401	1424	1396	1440	1403	1430	1399
342	1396	1433	1402	1428	1395	1437	1402	1430	1400
343	1396	1434	1402	1427	1393	1441	1403	1432	1400
344	1397	1435	1402	1427	1383	1443	1405	1433	1401
345	1410	1469	1423	1437	1379	1477	1435	1457	1412
346	1411	1468	1423	1438	1380	1476	1431	1457	1413
347	1412	1470	1424	1440	1380	1475	1430	1458	1414
348	1414	1473	1428	1439	1383	1481	1437	1461	1417
349	1414	1473	1428	1439	1385	1478	1433	1461	1417
350	1415	1472	1428	1439	1383	1482	1438	1461	1418
351	1416	1474	1430	1441	1394	1482	1438	1462	1419
352	1418	1476	1431	1442	1391	1486	1442	1465	1421
353	1429	1488	1439	1462	1410	1494	1445	1480	1431
354	1432	1492	1443	1462	1410	1498	1449	1483	1434
355	1433	1493	1445	1462	1410	1497	1450	1484	1434
356	1435	1495	1446	1464	1411	1501	1453	1486	1437
357	1438	1500	1451	1466	1411	1503	1455	1489	1438
358	1440	1500	1453	1465	1410	1504	1456	1489	1440
359	1441	1501	1455	1465	1410	1505	1458	1490	1441
360	1441	1502	1453	1467	1412	1506	1457	1492	1441
361	1454	1509	1459	1491	1430	1518	1467	1505	1457
362	1455	1509	1459	1492	1432	1518	1467	1506	1458
363	1455	1512	1461	1493	1432	1519	1468	1508	1459
364	1455	1511	1463	1491	1431	1518	1469	1507	1459
365	1456	1509	1458	1494	1435	1520	1471	1508	1461
366	1457	1512	1463	1494	1435	1522	1472	1509	1462
367	1458	1512	1463	1496	1437	1524	1474	1510	1463
368	1459	1512	1462	1496	1437	1522	1475	1511	1465

#	PAW	6-31G**		def2-SVP		pob-TZVP		def2-TZVP	
	PBE	PBE0	PBE	PBE0	PBE	PBE0	PBE	PBE0	PBE
369	1495	1564	1501	1556	1486	1563	1500	1558	1497
370	1496	1563	1501	1555	1486	1565	1503	1558	1498
371	1497	1567	1503	1559	1489	1565	1501	1560	1499
372	1497	1567	1503	1558	1488	1566	1503	1561	1499
373	1501	1570	1507	1561	1492	1570	1508	1563	1503
374	1502	1570	1506	1562	1492	1570	1509	1564	1504
375	1503	1572	1509	1565	1495	1572	1509	1566	1505
376	1508	1577	1513	1569	1500	1576	1512	1572	1509
377	1548	1623	1554	1607	1529	1625	1556	1615	1550
378	1550	1625	1556	1609	1531	1624	1554	1617	1552
379	1553	1629	1559	1614	1534	1632	1562	1621	1556
380	1554	1632	1560	1619	1538	1634	1562	1624	1557
381	1557	1631	1562	1613	1535	1633	1564	1623	1560
382	1559	1635	1565	1618	1539	1636	1565	1626	1562
383	1565	1638	1568	1621	1541	1642	1570	1631	1568
384	1577	1650	1579	1635	1553	1660	1590	1645	1580
385	1589	1675	1601	1667	1588	1670	1586	1669	1591
386	1590	1678	1603	1669	1590	1671	1585	1670	1592
387	1593	1675	1601	1667	1588	1675	1592	1670	1595
388	1595	1678	1604	1670	1591	1678	1595	1672	1597
389	1598	1676	1604	1667	1589	1676	1595	1671	1601
390	1603	1678	1605	1668	1589	1685	1609	1673	1605
391	1603	1680	1608	1670	1591	1683	1600	1675	1606
392	1604	1695	1620	1697	1616	1679	1594	1684	1607
393	1606	1693	1617	1694	1613	1687	1616	1682	1608
394	1608	1694	1619	1696	1615	1689	1615	1685	1611
395	1609	1683	1611	1672	1593	1690	1619	1678	1611
396	1612	1701	1623	1702	1616	1693	1620	1691	1614
397	1613	1697	1621	1698	1616	1697	1621	1688	1615
398	1613	1702	1624	1703	1621	1695	1619	1691	1616
399	1615	1696	1620	1696	1613	1695	1620	1688	1617
400	1616	1698	1622	1698	1615	1700	1625	1690	1619
401	1619	1728	1640	1729	1635	1699	1627	1703	1621
402	1621	1732	1643	1733	1638	1703	1625	1707	1623
403	1622	1732	1643	1733	1638	1705	1630	1707	1624
404	1623	1727	1641	1728	1636	1703	1628	1703	1625
405	1624	1734	1645	1733	1638	1702	1627	1710	1625
406	1625	1739	1648	1738	1642	1707	1630	1714	1627
407	1635	1747	1656	1749	1650	1714	1636	1725	1638
408	1645	1762	1669	1764	1666	1724	1638	1738	1648
409	2990	3067	2981	3053	2948	3076	2992	3066	2988
410	2990	3068	2982	3054	2949	3076	2992	3067	2988
411	2990	3067	2982	3053	2948	3076	2992	3066	2988
412	2990	3068	2982	3054	2948	3076	2992	3067	2988
413	2990	3068	2983	3052	2949	3076	2991	3067	2989
414	2990	3068	2982	3054	2948	3076	2992	3067	2989

#	PAW	6-31G**		def2-SVP		pob-TZVP		def2-TZVP	
	PBE	PBE0	PBE	PBE0	PBE	PBE0	PBE	PBE0	PBE
415	2990	3068	2982	3054	2948	3076	2992	3067	2989
416	2990	3068	2982	3054	2948	3076	2992	3067	2989
417	3013	3395	3106	3351	3075	3243	2949	3323	2997
418	3020	3398	3110	3355	3080	3250	2958	3329	3004
419	3022	3399	3115	3356	3083	3250	2959	3329	3007
420	3022	3403	3113	3359	3081	3254	2960	3332	3007
421	3023	3406	3115	3362	3082	3255	2961	3333	3008
422	3038	3406	3126	3365	3095	3263	2977	3340	3023
423	3040	3416	3129	3373	3097	3268	2979	3344	3024
424	3060	3428	3148	3386	3115	3288	3006	3359	3046
425	3063	3149	3058	3148	3033	3153	3063	3144	3063
426	3063	3150	3059	3148	3033	3153	3063	3145	3063
427	3063	3150	3059	3148	3033	3153	3064	3145	3063
428	3063	3150	3059	3148	3033	3153	3063	3145	3063
429	3063	3150	3059	3148	3033	3154	3064	3145	3063
430	3063	3150	3059	3148	3033	3153	3063	3145	3063
431	3063	3150	3059	3148	3033	3154	3064	3145	3063
432	3064	3149	3058	3148	3033	3153	3063	3144	3063
433	3094	3180	3088	3179	3064	3187	3096	3176	3095
434	3094	3180	3089	3179	3064	3187	3096	3176	3095
435	3094	3180	3088	3179	3064	3187	3096	3176	3095
436	3094	3180	3088	3179	3064	3187	3096	3176	3096
437	3094	3180	3089	3179	3065	3187	3097	3176	3095
438	3094	3181	3089	3180	3064	3188	3097	3177	3096
439	3094	3180	3089	3179	3064	3187	3097	3176	3096
440	3094	3180	3088	3179	3064	3187	3097	3176	3096
441	3115	3202	3111	3186	3074	3200	3109	3197	3113
442	3115	3202	3112	3186	3074	3200	3109	3197	3113
443	3115	3202	3111	3186	3075	3201	3110	3197	3113
444	3115	3202	3110	3186	3074	3201	3110	3197	3113
445	3116	3203	3112	3186	3074	3201	3111	3198	3114
446	3116	3203	3111	3186	3074	3201	3111	3198	3114
447	3116	3203	3110	3186	3075	3202	3111	3198	3114
448	3116	3203	3111	3186	3075	3202	3111	3198	3114
449	3131	3224	3133	3204	3093	3220	3130	3217	3132
450	3131	3224	3133	3204	3093	3220	3130	3217	3132
451	3131	3225	3133	3205	3093	3220	3130	3217	3132
452	3131	3225	3133	3204	3093	3220	3130	3218	3132
453	3132	3225	3133	3204	3093	3221	3130	3218	3133
454	3132	3225	3133	3204	3093	3221	3130	3218	3133
455	3132	3225	3133	3204	3093	3221	3130	3218	3133
456	3132	3225	3134	3204	3092	3221	3130	3218	3133
457	3148	3233	3144	3219	3110	3233	3146	3232	3152
458	3148	3233	3144	3219	3109	3233	3146	3232	3152
459	3148	3233	3143	3219	3109	3232	3146	3232	3152
460	3148	3233	3143	3219	3110	3233	3146	3232	3152

#	PAW	6-31G**		def2-SVP		pob-TZVP		def2-TZVP	
	PBE	PBE0	PBE	PBE0	PBE	PBE0	PBE	PBE0	PBE
461	3148	3233	3144	3219	3110	3233	3146	3232	3152
462	3148	3233	3143	3219	3110	3233	3146	3232	3152
463	3148	3233	3144	3219	3110	3233	3146	3232	3152
464	3148	3233	3144	3219	3110	3233	3146	3232	3152
465	3179	3276	3182	3253	3139	3261	3171	3271	3185
466	3179	3276	3182	3253	3139	3261	3171	3271	3185
467	3179	3276	3182	3253	3139	3261	3171	3271	3185
468	3179	3276	3182	3253	3139	3261	3171	3271	3185
469	3179	3276	3182	3253	3140	3262	3172	3272	3185
470	3179	3276	3182	3253	3139	3261	3172	3272	3185
471	3179	3276	3182	3253	3138	3261	3171	3271	3185
472	3179	3276	3182	3253	3139	3261	3172	3271	3185
473	3308	3477	3320	3453	3293	3408	3247	3469	3295
474	3309	3475	3320	3451	3293	3408	3249	3469	3296
475	3311	3480	3322	3456	3295	3412	3250	3472	3297
476	3311	3478	3322	3454	3295	3411	3250	3471	3297
477	3313	3482	3324	3457	3297	3413	3253	3472	3299
478	3315	3480	3326	3457	3298	3415	3255	3474	3301
479	3316	3480	3327	3456	3299	3415	3257	3474	3302
480	3318	3489	3329	3465	3301	3421	3259	3479	3305

References

(1) Dovesi, R. et al. The CRYSTAL code, 1976–2020 and beyond, a long story. *The Journal of Chemical Physics* **2020**, *152*, 204111.

(2) Dovesi, R. et al. CRYSTAL23. Gruppo di Chimica Teorica, Dipartimento di Chimica, Università di Torino, 2023.

(3) Giannozzi, P. et al. Advanced capabilities for materials modelling with Quantum ESPRESSO. *Journal of Physics: Condensed Matter* **2017**, *29*, 465901.

(4) Giannozzi, P. et al. QUANTUM ESPRESSO: a modular and open-source software project for quantum simulations of materials. *Journal of Physics: Condensed Matter* **2009**, *21*, 395502.

(5) Dal Corso, A. Pseudopotentials periodic table: From H to Pu. *Computational Materials Science* **2014**, *95*, 337–350.

(6) Zhu, Z.; Park, J.; Sahasrabuddhe, H.; Ganose, A. M.; Chang, R.; Lawson, J. W.; Jain, A. A high-throughput framework for lattice dynamics. *npj Computational Materials* **2024**, *10*, 258.

(7) Pascale, F.; Tosoni, S.; Zicovich-Wilson, C.; Ugliengo, P.; Orlando, R.; Dovesi, R. Vibrational spectrum of brucite, Mg(OH)2: a periodic ab initio quantum mechanical calculation including OH anharmonicity. *Chemical Physics Letters* **2004**, *396*, 308–315.

(8) Dunning Jr, T. H. Gaussian basis sets for use in correlated molecular calculations. I. The atoms boron through neon and hydrogen. *The Journal of Chemical Physics* **1989**, *90*, 1007–1023.

(9) Kendall, R. A.; Dunning Jr, T. H.; Harrison, R. J. Electron affinities of the first-row atoms revisited. Systematic basis sets and wave functions. *The Journal of Chemical Physics* **1992**, *96*, 6796–6806.

(10) Daga, L. E.; Civalleri, B.; Maschio, L. Gaussian Basis Sets for Crystalline Solids: All-Purpose Basis Set Libraries vs System-Specific Optimizations. *Journal of Chemical Theory and Computation* **2020**, *16*, 2192–2201.

(11) Lee, J.; Feng, X.; Cunha, L. A.; Gonthier, J. F.; Epifanovsky, E.; Head-Gordon, M. Approaching the basis set limit in Gaussian-orbital-based periodic calculations with transferability: Performance of pure density functionals for simple semiconductors. *The Journal of Chemical Physics* **2021**, *155*, 164102.

(12) Grimme group Package Manual for gCP Version 2.01. Universität Bonn, 2015.

(13) Sure, R.; Brandenburg, J. G.; Grimme, S. Small Atomic Orbital Basis Set FirstPrinciples Quantum Chemical Methods for Large Molecular and Periodic Systems: A Critical Analysis of Error Sources. *ChemistryOpen* **2016**, *5*, 94–109.

(14) Wilson, C. Variable temperature study of the crystal structure of paracetamol (p-hydroxyacetanilide), by single crystal neutron diffraction. *Zeitschrift für Kristallographie - Crystalline Materials* **2000**, *215*, 693–701.

(15) Druzhbin, D. A.; Drebushchak, T. N.; Minkov, V. S.; Boldyreva, E. V. Crystal structure of two paracetamol polymorphs at 20 K: A search for the structure-property relationship. *Journal of Structural Chemistry* **2015**, *56*, 317–323.

(16) Tsapatsaris, N.; Kolesov, B. A.; Fischer, J.; Boldyreva, E. V.; Daemen, L.; Eckert, J.; Bordallo, H. N. Polymorphism of Paracetamol: A New Understanding of Molecular Flexibility through Local Methyl Dynamics. *Molecular Pharmaceutics* **2014**, *11*, 1032–1041.

(17) Kapil, V.; Engel, E.; Rossi, M.; Ceriotti, M. Assessment of Approximate Methods for Anharmonic Free Energies. *Journal of Chemical Theory and Computation* **2019**, *15*, 5845–5857.

(18) Nanubolu, J. B.; Burley, J. C. Investigating the Recrystallization Behavior of Amorphous Paracetamol by Variable Temperature Raman Studies and Surface Raman Mapping. *Molecular Pharmaceutics* **2012**, *9*, 1544–1558.

(19) Kruse, H.; Grimme, S. A geometrical correction for the inter- and intra-molecular basis set superposition error in Hartree-Fock and density functional theory calculations for large systems. *The Journal of Chemical Physics* **2012**, *136*, 154101.

(20) Brandenburg, J. G.; Alessio, M.; Civalleri, B.; Peintinger, M. F.; Bredow, T.; Grimme, S. Geometrical Correction for the Inter- and Intramolecular Basis Set Superposition Error in Periodic Density Functional Theory Calculations. *The Journal of Physical Chemistry A* **2013**, *117*, 9282–9292.

(21) Boys, S. F.; Bernardi, F. The calculation of small molecular interactions by the differences of separate total energies. Some procedures with reduced errors. *Molecular Physics* **1970**, *19*, 553–566.

(22) Beyer, T.; Day, G. M.; Price, S. L. The Prediction, Morphology, and Mechanical Properties of the Polymorphs of Paracetamol. *Journal of the American Chemical Society* **2001**, *123*, 5086–5094.

(23) Adhikari, K.; Flurchick, K. M.; Valenzano, L. Volumetric influence on the mechanical behavior of organic solids: The case of aspirin and paracetamol addressed via dispersion corrected DFT. *Chemical Physics Letters* **2015**, *630*, 44–50.

(24) Rossi, M.; Gasparotto, P.; Ceriotti, M. Anharmonic and Quantum Fluctuations in Molecular Crystals: A First-Principles Study of the Stability of Paracetamol. *Physical Review Letters* **2016**, *117*, 115702.

(25) Becket, G.; Beech-Brandt, J.; Leach, K.; Simpson, Z. P. A.; Turner, A.; Whiting, A. ARCHER2 Service Description. *Zenodo* **2024**,
